# A review of mechanisms of resistance to immune checkpoint inhibitors and potential strategies for therapy

**DOI:** 10.20517/cdr.2020.11

**Published:** 2020-06-18

**Authors:** Yu Fujiwara, Arjun Mittra, Abdul Rafeh Naqash, Naoko Takebe

**Affiliations:** ^1^Developmental Therapeutics Clinic, Division of Cancer Treatment and Diagnosis, National Cancer Institute, Bethesda, MD 20892, USA.; ^2^Department of Medical Oncology, The Cancer Institute Hospital of Japanese Foundation for Cancer Research, Tokyo 1358550, Japan.; ^3^Department of Medical Oncology, Musashino Red Cross Hospital, Tokyo 1800023, Japan.; ^*^These authors contributed equally to this work.

**Keywords:** Immunotherapy resistance, tumor microenvironment, PD-1, PD-L1, CTLA-4

## Abstract

Immune checkpoint inhibitors (ICIs) have revolutionized the treatment of cancer over the last decade, bringing about a paradigm shift in systemic cancer therapy away from traditional cytotoxic and targeted therapies. While some patients have dramatic treatment responses, it is sobering to note that most tumors are either resistant at the outset, or develop resistance after initial response. A major area of translational and clinical research is in identifying therapeutic strategies to overcome resistance to ICIs. We have performed an in-depth review of the different mechanisms of resistance and potential avenues to overcome resistance through rationally designed combination treatment with ICIs.

## Introduction

Cytotoxic chemotherapy and targeted molecular therapy have been the mainstay of treatment for metastatic cancers for several decades. While significant improvement has been made over this period, the vast majority of metastatic tumors remain incurable due to inherent genomic instability, which allows them to develop resistance to these therapies^[1]^. Recent advances in cancer immunology and immunotherapy have resulted in a significant paradigm shift, allowing a subset of patients to see sustained treatment responses and some advanced cancers were even made “curable”^[2]^.

The foundations of the modern era of tumor immunology were laid in the late nineteenth century when William Coley witnessed the regression of malignant tumors in patients who developed a bacterial infection^[3,4]^. However, with the rapid progress of chemotherapy and radiation therapy in the first half of the twentieth century, the development of immunotherapy was overlooked. During the latter half of the century, tumor immunology was harnessed to treat certain cancers with the development of the BCG vaccine, interferon, and interleukin-2^[5]^. Our understanding of T cell anti-tumor responses has come a long way through incremental steps of elucidating the intricate interaction between cancer cells, T cells, antigen-presenting cells, immunosuppressive cells, and other immune mechanisms^[6]^. The dynamic interplay between inhibitory and stimulatory signals on T cells modulates the degree of immune activation to allow tolerance to self-antigens (inhibitory) while mounting an adaptive immune response to foreign antigens (stimulatory)^[7]^. An essential mechanism of inhibitory stimuli is through immune checkpoints, which help to control the inflammatory response after T cell activation and protect normal cells from T cell mediated cytotoxicity. This protective mechanism, however, may be a disadvantage for cancer cells as the T cell immune response effect towards cancer cells is eliminated within the tumor microenvironment (TME)^[7]^.

The first immune checkpoint to be characterized is the cytotoxic T lymphocyte-associated protein 4 (CTLA-4) receptor, identified on activated T cells and regulatory T cells (Tregs)^[8]^. CTLA-4 competes with CD28 for the B7 ligand, inhibiting T cell proliferation and IL-2 secretion, and has been shown to inhibit tumor cell proliferation^[9]^. Another checkpoint receptor expressed by activated T cells is programmed death 1 (PD-1) and its ligand programmed cell death ligand 1 (PD-L1)^[10]^. The expression of PD-L1 on tumor cells can serve as a potent mechanism for escaping host immune responses^[11]^ and the blockade of the PD-1 and PD-L1 axis has been shown to lead to an anti-tumor immune response^[12,13]^.

**Figure 1:**
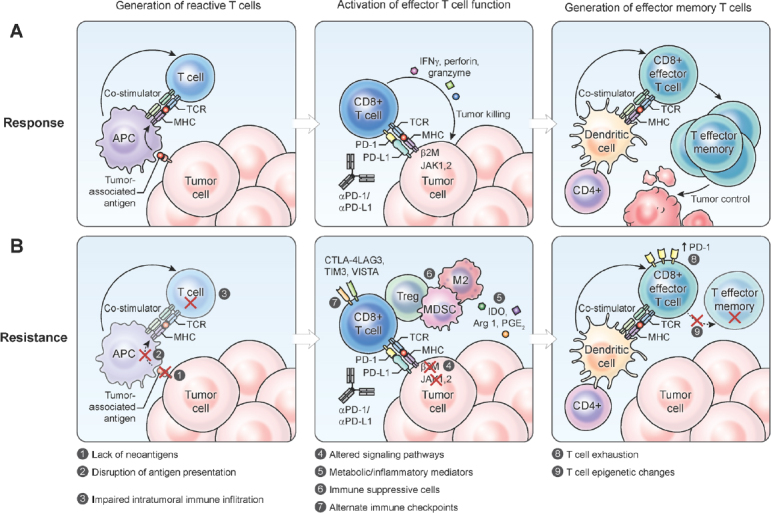
Steps involved in the generation of an effective tumor-directed T cell response through the formation of tumor reactive T cells, the activation of effector T cell function and the formation of effector memory T cells (A); resistance to immune checkpoint inhibitors may occur due to multiple different factors within the tumor cell or the tumor microenvironment (B). MHC: major histocompatibility complex; PD-1: programmed death 1; PD-L1: programmed cell death ligand 1; MDSC: myeloid-derived suppressor cell; IDO: Indoleamine 2,3-dioxygenase; APC: antigen-presenting cell

The CTLA-4 inhibitor ipilimumab was approved by the US Food and Drug Administration (FDA) in 2011 for metastatic malignant melanoma, serving as the first new agent in this class of ICIs^[14]^. In 2014, the anti-PD-1 antibodies, pembrolizumab and nivolumab were approved by the FDA for advanced melanoma and additional agents in these classes were also approved. Currently, three anti-PD-1 antibodies (pembrolizumab, nivolumab and cemiplimab) and three anti-PD-L1 antibodies (atezolizumab, durvalumab and avelumab) have been approved by the FDA for the treatment of various types of cancer. Despite thousands of patients treated with ICIs - both on and off trial - the sobering reality is that while some patients have had dramatic and sustained responses, the overall treatment response rate to these agents is usually low and some patients who do have tumor regression initially, start losing that response over time^[15]^. These findings would suggest the existence of resistance mechanisms to checkpoint inhibitors: primary resistance in tumors that have no response, and secondary resistance in those that responded initially but lose that response over time. Resistance to ICIs can occur at different points in the T cell activation process [Figure 1].

As ICIs have become an established part of the oncologist’s armamentarium, a major focus of ongoing clinical and translational research is the identification of avenues to overcome resistance to these agents and thereby, broadening the cohort of patients with cancer who may benefit from them. Understanding the mechanisms of resistance is thus crucial to this approach of personalizing the delivery of immunotherapy. In this article, we review the different mechanisms of resistance to ICIs. Additionally, we explore potential strategies to overcome resistance and improve clinical outcomes in patients with cancer.

## Mechanisms of resistance

**Figure 2:**
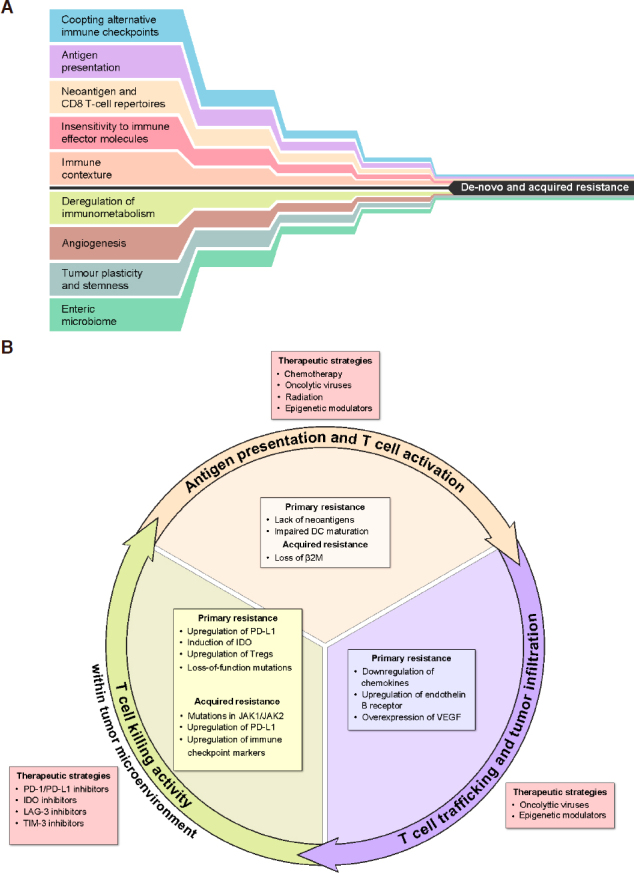
Mechanisms that may either alone, or in combination lead to de novo or acquired resistance to immune checkpoint inhibition (A); Primary and acquired mechanisms of resistance at different points in the cancer immunity cycle and potential therapeutic strategies to overcome them (B). PD-1: programmed death 1; PD-L1: programmed cell death ligand 1; IDO: Indoleamine 2,3-dioxygenase; VEGF: vascular endothelial growth factor

Resistance can be primary, in tumors that have never responded to ICIs, or acquired, where resistance occurs after a period of initial response. Resistance can also be classified as intrinsic or extrinsic to tumor cells. Intrinsic resistance is observed when cancer cells alter processes that are relevant to immune recognition, cell signaling pathways, gene expression, and DNA damage responses. Extrinsic resistance occurs external to tumor cells through immunological and non-immunological interaction among T cells, macrophages, myeloid-derived suppressor cells, immune checkpoints, enzymes, angiogenesis, and the microbiome^[6,16-18]^ [Figure 2A].

## Mechanisms of intrinsic resistance

### Lack of neoantigens

Tumor antigen-specific T cells may often be present within tumors without mounting an effective immune response^[19,20]^. This is more commonly seen in tumors that do not have a significantly increased tumor mutation burden (TMB) and do not express neoantigens that would stimulate a focused CD8+ T cell response against them. This absence of T cells that can recognize unique tumor antigens makes these tumors nonresponsive to ICI therapy^[21]^. Conversely, genetic or epigenetic changes in tumor cells could theoretically lead to acquired resistance after an initial response^[1]^.

Cancers with high immunogenicity which typically accompanies high TMB such as melanoma, renal cell carcinoma, and non-small cell lung cancer are known to be sensitive to immunotherapy, while in contrast, those such as prostate or pancreatic cancer with low immunogenicity tend to be minimally responsive to ICI therapy^[20,22-24]^. Renal cell carcinoma has a relatively low TMB and is a noteworthy exception, highlighting the presence of other factors contributing to its response to ICI^[25]^. Within the same cancer histology, the degree of the mutational load is also associated with variations in clinical response^[22,26]^.

Intracellular antigens are presented on the cell surface through the major histocompatibility complex (MHC), and disruption of such antigen presentation can result in resistance of the tumor to T cell mediated cytotoxicity. The antigen presentation mechanism may also be altered through mutations interfering with the transporter for antigen presentation (TAP), or within the structure of the MHC itself^[27]^. Moreover, alterations in the antigen-presenting machinery, beta 2 microglobulin (B2M), or the MHC itself may make cancer cells avoid presenting antigens on the surface^[28,29]^. Several studies show that downregulation of MHC class I allowed tumor cells to escape from immune surveillance^[30,31]^. Alterations or mutations in B2M, which lead to instability of the MHC, have been shown to form resistance to immunotherapy. Truncating alterations, loss of heterozygosity, frameshift mutations, and loss of function alterations in B2M were found in human tumor cells that showed resistance to ICI^[32,33]^. Loss of function of B2M led to the dysfunction of MHC class I, resulting in an escape from PD-1 inhibition^[34]^. Therefore, the genetic deficiency of B2M may cause a lack of tumor cell recognition by T cells because of the loss of MHC class I folding and transport to the cell surface.

Other than alterations or mutations in the MHC and its structure, the function of mismatch repair (MMR) is key to predicting sensitivity or resistance to ICI. Flaws in DNA replication of normal cells are usually recognized and corrected by the MMR system. High numbers of somatic mutations are seen in MMR-deficient tumors. One study showed that MMR-deficient solid tumors had somatic mutations approximately 25 times more than MMR-proficient tumors^[35]^. Also, the defect of MMR leads to the accumulation of erroneous genetic products in microsatellites, resulting in a status called microsatellite instability (MSI). MSI-high status was also found to produce more neoantigens and shown to work as a predictive marker for ICIs in many types of cancers^[24,35,36]^. Therefore, MSI-high status and MMR deficiency are valuable predictive biomarkers for ICI administration. However, there are multiple resistance mechanisms involved with neoantigen presentation as discussed here and it is important to identify biomarkers to evaluate the neoantigen load and response to ICI therapy.

### Epigenetic changes in cancer cells

Epigenetic modification such as methylation, histone modification, and silencing in cancer cells may alter the expression of immune-related genes, leading to changes in antigen processing, antigen presentation, immune evasion, and T cell exhaustion^[37,38]^. For example, methylation of the MHC class I transactivator known as the NOD-like receptor family, caspase recruitment domain containing 5 (NLRC5), caused suppression of MHC class I, led to poor antigen presentation, and was linked to poor survival^[39]^. Among cancers with low MHC class I antigen processing pathway (MHC-I APP), histone modifications were seen in MHC-I APP gene promotors, leading to transcriptional silencing and decreased expression of MHC class I, causing loss of antigen presentation and immune evasion^[40]^. In prostate cancer, epigenetic silencing of JAK1 was seen and associated with insensitivity to Interferon (IFN) gamma, resulting in immune evasion and resistance to immunotherapy^[41]^.

Thus, agents like DNA methyltransferase (DNMT) inhibitors and histone deacetylase (HDAC) inhibitors can cause expression of immune-related genes and reverse immune suppression by increasing the expression of antigens, related molecules, and improvement of antigen processing, presentation, and function of chemokines^[42]^. DNMT inhibitors like decitabine and azacytidine are candidates for combination therapy with ICIs. Decitabine may directly enhance cytolytic activity through the increased production of IFN-gamma by T cell activation^[43]^. De novo DNA methylation is known to contribute to T cell exhaustion and decitabine has the potential to reverse this exhausted state and restore a T cell abundant TME^[44]^. Decitabine also has a role in increasing the expression of new, MHC class I molecules and several murine cancer-testis antigens, suggesting the possibility of improving the efficacy of immunotherapy^[45]^. Entinostat, an HDAC inhibitor, has been shown to neutralize myeloid-derived suppressor cells and suppress Tregs, which enhances immunotherapy including IL-2, vaccines and PD-1 inhibitors in murine models of lung, renal cell carcinoma, and prostate cancer^[46,47]^. This agent may alter resistance in breast and pancreatic cancers, making them more responsive to immunotherapy by modulating myeloid-derived suppressor cells (MDSCs) surrounding tumor cells^[48]^. Findings from ENCORE 601, a phase Ib/II trial showed that pembrolizumab may derive benefit from the addition of entinostat in NSCLC and melanoma patients. In this trial, a reduced number of circulating MDSCs from baseline were found to be associated with clinical response both in NSCLC and melanoma, which were consistent with preclinical results^[48-51]^. Another phase I/Ib study using pembrolizumab and vorinostat, an HDAC inhibitor, showed preliminary antitumor activity, although no difference in the change of MDSCs was found between responders and non-responders. The high level of stromal CD8+ T cells was associated with response in this study and further investigation to define the mechanism of efficacy from this combination therapy is necessary^[52]^. Overall, these preclinical and clinical results suggest that epigenetic changes are responsible for resistance to immunotherapy and epigenetic modifiers are strong candidates to overcome primary resistance.

### Alteration of signaling pathways

There is an increasing body of evidence that the activation of different oncogenic cell signaling pathways can impair local anti-tumor immune response and are relevant across all the different stages of carcinogenesis^[53,54]^.

The RAS/RAF/mitogen-activated protein kinase (MAPK) pathway functions to transduce signals from the extracellular space into the nucleus where it affects cell proliferation, division, and differentiation^[55]^. Dysregulation of this signaling pathway is implicated in malignant transformation^[56]^. The MAPK pathway exerts an inhibitory influence on T cell recruitment and function by yielding vascular endothelial growth factor (VEGF) and IL-8, leading to a T cell-poor environment^[57]^. Also, activation of the MAPK pathway through mutations in EGFR and KRAS, and EML4-ALK fusion may upregulate PD-L1 expression, suggesting that the MAPK pathway creates the immunosuppressive environment and forms resistance to immunotherapy^[58-60]^. Conversely, the inhibition of the MAPK pathway promotes CD8+ T cell activation and infiltration within the TME and induces the expression of tumor antigens through enhancing MHC class I expression^[61-63]^.

The PI3K/AKT signaling pathway is a critical intracellular signaling pathway that regulates cell proliferation and may be abnormally activated in cancer^[56,64]^. Loss of PTEN enhances PI3K signaling, leading to the production of immunosuppressive cytokines such as VEGF and decreasing T cell infiltration into the TME^[65]^. PTEN mutations are also potential mediators of resistance to ICI therapy^[66]^. Wnt signaling is an essential regulatory pathway that governs many cellular processes such as proliferation, migration, and stemness. Aberrations in wnt signaling are increasingly being identified as an important mechanism of oncogenesis^[67]^. Furthermore, tumor-intrinsic activating β-catenin signaling can cause T cell exclusion in melanoma and resistance to ICI therapy^[54]^. A recent study analyzing The Cancer Genome Atlas (TCGA) showed a three-fold enrichment of β-catenin mutations in T cell-inflamed tumors compared to non-inflamed tumors^[68]^. These findings support the future development of inhibitors of this pathway for recruiting immune cell infiltration and improving the outcomes of immunotherapy.

### Regulation of IFN

IFN-gamma is one of the T cell effector cytokines and is known to help inhibit tumor cell growth by suppressing cell proliferation, facilitating apoptosis in cancer cells, and upregulating tumor antigen presentation on beta-2 microglobulin through the JAK-STAT signaling pathway. Persistent IFN-gamma stimulation renders susceptible melanoma cells resistant to immunotherapy^[69]^; while the increased frequency of mutations in the IFN-gamma pathway genes (IFN-gamma receptor 1 and 2, JAK2, and interferon regulatory factor 1) were detected through the analysis of tumor cells in patients who did not respond to anti-CTLA-4 therapy^[70]^. Additionally, JAK1/2 mutation leads to primary resistance to PD-1/PD-L1 blockade treatment through the loss of IFN-gamma induced expression of MHC class I and PD-L1, phosphorylation of the STAT transcription factor, and IFN-gamma mediated growth arrest^[71]^. Thus, tumor cells can escape the effects of IFN-gamma by downregulating or mutating molecules involved in the IFN-gamma signaling pathway.

## Mechanisms of extrinsic resistance

Extrinsic factors that contribute to the formation of resistance to immunotherapy are mainly involved with the TME. Other causes are inhibitory immune checkpoints, cytokines, factors associated with angiogenesis, and other remote factors such as the microbiome.

### Absence of T cells and the STING pathway

Many factors intricately interact with each other within tumor cells and in the TME. Intrinsic factors that cause a decrease in intratumoral T cell infiltration include epigenetic changes and intracellular signaling pathways. However, dendritic cells (DCs) or other antigen-presenting cells (APCs) are known to play important roles in the infiltration of T cells into the TME [Figure 1]. The stimulator of interferon genes (STING) signaling pathway significantly contributes to innate immune sensing of immunogenic tumors^[72]^. The upregulation of this pathway results in the activation of antigen-presenting cells and priming of CD8+ T cells against tumors. It was shown that STING-deficient mice cannot provoke a sufficient T cell response against tumor cells due to insufficient recruitment and activation of DCs. Moreover, the inhibition of both CTLA-4 and PD-1/PD-L1 interactions is not effective in a STING-deficient mouse model^[72]^. Downstream of the STING pathway is IFN-beta, which is essential in presenting tumor antigen to CD8+ T cells. There are multiple reports to establish the rationale for using STING-agonists for cancer treatment as potential candidates to overcome immunotherapy resistance^[73-77]^.

Intratumoral administration of a STING agonist such as cyclic diguanylate monophosphate (CDG) and synthetic cyclic dinucleotide (CDN) suppresses tumor growth with enhanced type I IFN signaling, chemokines including CXCL10 and CCL5, and T cell migration^[74,78]^. Moreover, the combination of intratumoral CDG injection with a systemic checkpoint inhibitor potentiated survival benefit in a mouse model with castrate-resistant prostate cancer by enhancing intratumoral ratios of CD8+ T cells to Tregs, macrophages, and MDSCs synergistically. Single-agent CDG failed to elicit the abscopal effect in this study, suggesting that combination immunotherapy is necessary to provide systemic benefit^[77]^. Currently, multiple strategies have been implemented to enhance the systemic efficacy of treatment using the STING pathway. For instance, nanoparticles or liposomal delivery was shown to enhance immune efficacy^[79,80]^, and a STING agonist conjugated with antibodies as antibody-drug conjugates has been developed^[81]^. Several clinical trials are ongoing to target the STING pathway to overcome immunotherapy resistance (NCT04096638) (NCT03010176).

### Exosomal PD-L1

Ordinarily, PD-L1 on the surface of tumor cells interacts with PD-1 to suppress the immune reaction around the tumor environment. However, PD-L1 has also been detected on the surface of extracellular vesicles (EV) generated from tumor cells, and this EV PD-L1 is associated with the suppression of immunity against tumor and tumor progression^[82,83]^. Additionally, a particular form of EV PD-L1, exosomal PD-L1, is presumably helpful in predicting the efficacy of anti-PD-1 therapy^[84]^. A study showed the genetic blockage of this exosomal PD-L1 facilitates activation of T cells in the draining lymph node and exosomal PD-L1-deficient tumor cells suppress tumor cell proliferation. Thus, exosomal PD-L1 seems to contribute to resistance to ICI and could be a possible molecular target to regain anti-tumor immunity^[85]^.

### Immunosuppressive cells

#### Tregs

Regulatory T cells (Tregs) are known to suppress effector T cells and APCs, maintaining immune status and self-tolerance^[86-88]^. They do so by three principal mechanisms: consuming IL-2 through its receptor CD25, downregulating costimulatory signals to effector T cells through the binding of CTLA-4 and CD80/86, and direct inhibition by secreting inhibitory cytokines including IL-35, IL-10 and transforming growth factor β (TGF-β) or inducing apoptosis by producing perforin and granzyme^[89-91]^. In addition to these known mechanisms, the increased levels of Tregs and decreased ratio of effector T cells to Tregs in the TME is associated with a poor prognosis in human tumors^[92]^. Conversely, the high levels of FoxP3 protein, which was relatively specific for Tregs prior to the initiation of ipilimumab treatment for melanoma patients, were related to better clinical outcomes^[93]^. Therefore, Tregs are indeed a desirable target to overcome resistance to ICI therapy. Previous studies have found that anti-CTLA-4 antibody depletes tumor-infiltrating Tregs in the melanoma mouse model, which expressed granulocyte macrophage colony-stimulating factor and Fcγ receptor on macrophages was involved with depletion of Tregs by an anti-CTLA-4 antibody^[94]^. To overcome resistance to anti-CTLA-4 antibody monotherapy, a strategy for increasing intratumoral Treg depletion was attempted. The depletion of Tregs and greater antitumor efficacy were gained by an engineered Fc-region-modified anti-CTLA-4 antibody with high antibody-dependent cell-mediated cytotoxicity or adding Toll-like receptor 1/2 ligand to anti-CTLA-4 antibody in preclinical models^[95-97]^. These findings were meaningful in building a strategy for tumors with high relative expression of Tregs.

#### Myeloid-derived suppressor cells

Myeloid-derived suppressor cells (MDSCs) are one of the key mediators controlling the immune status in the TME^[98]^. They have multiple roles including the suppression of CD8+ T cells, angiogenesis, cell invasion, and metastasis in cancer cells^[99,100]^. A lower frequency of circulating MDSCs in patients with melanoma is associated with a higher response to ipilimumab^[101]^. Moreover, long-time melanoma survivors who were previously treated with ipilimumab had a decrease in MDSCs^[102]^. These clinical data suggest that MDSCs may be an essential biomarker to predict the efficacy of ICI and a potential target to overcome ICI resistance.

One potential method to target MDSCs is through the selective inactivation of macrophage PI3Kγ, which in turn stimulates an immune-stimulatory transcriptional program leading to CD8+ T cell activation and cytotoxicity. Additionally, PI3Kγ inhibition has been shown to synergize with ICI therapy, leading to tumor regression and increased survival in a mouse model^[103]^. Another function of PI3Kγ signaling is activating integrin α4 to promote myeloid cell adhesion and invasion into tumor cells. Indeed, integrin α4 was shown to promote MDSCs polarization, inhibit antitumor immunity, and facilitate tumor growth. Consequently, inhibition of PI3Kγ and integrin α4 was shown to be associated with the stimulation of DC, recruitment of CD8+ T cells, prevention of MDSCs accumulation, and reduction of immunosuppressive factors around tumor cells^[104]^. Moreover, anti-PD-L1 efficacy is strongly enhanced when treated with a selective inhibitor of PI3Kδ/γ through the inhibition of MSDCs^[105]^. Based on these data, several clinical trials using ICIs and PI3K inhibitors are currently underway. Recently, TNF-related apoptosis induced ligand-receptors (TRAIL-R) agonistic antibody opened up possibilities as a new treatment option for its apoptotic function and possibly as a selective inhibitor of MDSCs^[106]^.

The vitamin A derivative all-trans retinoic acid (ATRA), which is part of the standard treatment for acute promyelocytic leukemia (APL) is another compound that can be used for MDSCs suppression^[107]^. ATRA is known to induce differentiation of immature myelocytic cells and likewise, differentiate and decrease circular MDSCs. The combination of ATRA with different immunostimulatory agents has shown that ATRA can decrease the number of circulating MDSCs^[108-110]^.

#### Tumor-associated macrophages

According to the environment, whereever they migrate to, macrophages can be classified as M1 or M2. M2 macrophages possess tumorigenic features and macrophages that are educated in the TME differentiate into the specific form known as tumor-associated macrophages (TAMs). Increased levels of TAMs are known to be associated with metastasis and poor prognosis in human tumors. *In vivo*, TAMs capture the anti-PD-1 antibody from the T cell surface immediately after the anti-PD-1 antibody binds PD-1-positive tumor-infiltrating CD8+ T cells, causing resistance to the ICI^[111]^. For these reasons, targeting TAMs is a legitimate way to restrain tumor progression. Potential agents are CXCR4 inhibitors, CSF-1R inhibitors, an anti-macrophage receptor with collagenous structure (MARCO) antibody, and PD-1 inhibitor. CXCR4, a chemokine receptor, is a G protein-coupled receptor and usually expressed on monocytes, B cells, and naïve T cells in the peripheral blood^[112]^. CXCR4 has an important function in tumor metastasis, progression, and angiogenesis^[113]^. In chemotherapy-treated mouse tumors, TAMs which expressed CXCR4 were predominant cells in the TME and CXCR4 inhibitor monotherapy was found to block M2-like TAMs, resulting in the inhibition of tumor progression^[114]^. CSF-1R, a receptor for macrophage-colony stimulating growth factor, is another valuable molecule contributing to TAMs regulation. CSF-1R inhibitor showed tumor regression when combined with anti-PD-1 or CTLA-4 antibody and gemcitabine in pancreatic cancer models and showed synergy with adoptive cellular therapy in melanoma cell models^[115,116]^. MARCO is a scavenger receptor expressed on the surface of a group of suppressive TAMS, and a monoclonal antibody targeting it showed a response in breast and colorectal cancer, and melanoma models while facilitating the efficacy of anti-CTLA-4 antibody therapy^[116]^. PD-1 expressed on TAMs is associated with less phagocytosis against cancer cells and thus, PD-1/PD-L1 inhibition on TAMs increases macrophage phagocytosis^[117]^. This blockade also reduces tumor growth and improves survival in mouse models. This suggests that anti-PD-1/PD-L1 therapy is potentially useful not only by inhibiting T cell and tumor cell interaction but also by suppressing TAMs activity, leading to improvement in the efficacy of ICI^[117]^.

#### T cell exhaustion

T cells infiltrating into the TME have been demonstrated as having an active role in predicting the effectiveness of immunotherapy^[118]^. Other than mechanisms such as intracellular signaling pathways, epigenetic changes that lead to T cell exhaustion, there are additional factors that exclude the assembly of T cells and make the microenvironment invulnerable to cytotoxic T cells. As prior reports have demonstrated, abundant tumor infiltrating CD8+ T cells are crucial for determining sensitivity to immunotherapy^[119]^. Additionally, sufficient numbers of tumor-infiltrating lymphocytes correlate with a high rate of objective response in melanoma patients who undergo adoptive cell transfer treatment^[120]^. Constant T cell receptor stimulation with tumor-specific neoantigen induces an increased level of PD-1 expression on effector T cells, consequently leading them into an exhausted and ineffective state^[121]^. Increased expression of PD-1 in effector T cells correlates with poor prognosis, while the degree of PD-L1 expression may be related to the efficacy of ICI therapy^[121-123]^.

Recently, T cell dysfunction was shown to determine the efficacy of ICI therapy. Functional heterogeneity of T cells was observed during virus infection and around tumor cells and recent studies showed there were two distinct subsets of exhausted T cells - a progenitor subset and a terminally differentiated subset. A progenitor subset expresses T cell factor 1 (TCF1), PD-1, and a low level of TIM3. This subset accompanies stem cell-like properties, such as proliferative potential and ability to differentiate and memory T cell-like features. These progenitor T cells promoted tumor control in response to vaccination and ICI therapy, and an increased frequency of TCF1+PD-1+TIM3^low^ CD8+ T cells were associated with longer survival. On the other hand, a terminally differentiated subset was found to have exhausted properties with inhibitory receptor expression. This terminally differentiated subset (typically PD-1+TCF1-TIM3^high^ CD8+ T cells) has highly cytotoxic features but lacks long-term duration of response to ICI therapy. The differentiation of exhaustion in CD8 T cells was induced through epigenetic modification such as the transcriptional regulator TOX. These features suggest the strategy to increase the frequency of the progenitor subset which controls tumor control and can respond to ICI therapy is reasonable to overcome resistance to ICI. Moreover, evaluation of not only the numbers but also the specific state of exhaustion in tumor-infiltrating T cells could be a valuable predictor of immunotherapy response^[124-127]^. Along with effector T cells, memory T cells can also lapse into an exhausted condition in the context of continuous tumor antigen exposure. When compared to good responders, inadequate response to anti-PD-1 treatment is associated with fewer memory T cells, suggesting that weak memory T cell formation in the TME leads to insufficient efficacy of ICI therapy^[128]^. This finding suggests poor memory T cell formation in the TME leads to insufficient efficacy of cancer immunotherapy. To overcome this T cell exhaustion, the resurrection of effector T cell function is necessary. The strategy includes the inhibition of inhibitory immune checkpoints and the activation of costimulatory checkpoints expressed on the surface of T cells, leading to reactivation of T cell ability to attack tumor cells. These strategies are discussed separately in detail later.

#### Epithelial-mesenchymal transition

Recently, studies involving the epithelial-mesenchymal transition (EMT) have been attributed as an emerging mechanism of resistance to immunotherapy. EMT is the process that results in dedifferentiation of epithelial tumor cells to a mesenchymal phenotype with motile, invasive, and metastatic features due to tumor cell plasticity^[129]^. Several studies showed that EMT and tumor cell plasticity were associated with resistance to cancer therapy such as cytotoxic chemotherapy and molecular-targeting therapy^[130-132]^. In the process of EMT, cancer cells downregulate epithelial markers such as E-cadherin and upregulate mesenchymal markers including collagen, vimentin, and fibronectin, regulated by multiple molecules such as interleukin-8 (IL-8) and TGF-β, or transcription factors such as brachyury. Through EMT, mesenchymal-like tumors can acquire multiple immune escape mechanisms such as T cell exclusion in the TME, resistance to immune-mediated cytotoxicity by T cells and NK cells, and expression of PD-L1 on tumor cells^[129]^. Thus, factors involved with EMT are potentially targetable to overcome resistance to immunotherapy.

IL-8 has been shown to promote mesenchymal features in cancer cells with its receptors CXCR1 and CXCR2^[133]^. The overexpression of IL-8 is associated with poor clinical outcomes in multiple types of solid tumors^[134]^. TGF-β has also been shown to promote tumor cell plasticity through activation of multiple EMT transcriptional factors and immune suppression in the TME by T cell exclusion, resulting in resistance to immunotherapy^[135-137]^. TGF-β is well known to have multiple roles, from stemness to metastasis in cancer cells^[138]^. Its notable roles include the induction of fibrosis and angiogenesis as well as remodeling the TME through the suppression of T cell configuration^[139]^. TGF-β signaling activation in urothelial cancer fibroblasts triggering exclusion of CD8+ T cells from the tumor parenchyma has been identified as a cause for the lack of response to anti-PD-L1 therapy. This study also found that the combination therapy of TGF-β inhibitor with anti-PD-L1 treatment reduced TGF-β signaling in stromal cells, which enabled cytotoxic T cells to penetrate the tumors and converted the TME from “cold” to “hot” in a mouse model^[136]^. Recently, the use of bifunctional fusion proteins, which possesses anti-PD-L1 antibody linked to the extracellular domain of TGF-βR molecules, has been attempted with promising results in preclinical models and several early-phase clinical trials have been reported^[140-143]^.

Other than IL-8 and TGF-β, there are several molecules and transcriptional factors that have been developed to overcome ICI resistance and used in several clinical trials. For example, AXL, a member of the TAM receptor tyrosine kinase family, is an inducer of tumor cell plasticity and its expression increases tumor cell invasion and metastasis by promoting T cell exclusion^[144,145]^. AXL inhibitor or its pathway modulator is now used with ICIs including nivolumab, pembrolizumab, durvalumab, and avelumab^[129]^. Also, vaccine therapy that targets immunogenic peptides or epitopes of an EMT transcriptional factor may be a novel approach to overcome resistance. For example, vaccines directly targeting brachyury increased the number of CD8+ T cells and suppressed tumor growth in mouse models and human cancer cells^[146-149]^.

Based on these mechanisms of resistance and current trials, agents that modify tumor cell plasticity and reduce EMT would become treatment options for cancer patients.

#### Immune checkpoints other than PD-1 and CTLA-4

Other than PD-1 and CTLA-4, many immune checkpoints exist on the surface of T cells, and the interactions between them and ligands on tumor cells are potentially important to determine the clinical response of ICIs. The suppression of co-stimulatory checkpoints and activation of other inhibitory checkpoints is an important mechanism that renders immunotherapy inefficient. CD40, which belongs to the tumor necrosis factor receptor, is known to be expressed on tumor cells, and its ligand, CD40-L, on the surface of T cells^[150]^. CD40 stimulation converts the “cold” TME into an immunologically “rich” site, and the monotherapy of a CD40 agonist has shown tolerability in a phase I study^[151,152]^. A phase I study using the combination treatment of CD40 agonist with anti-CTLA-4 antibody, tremelimumab, for melanoma patients resulted in a 27.3% objective response rate and increased T cell activation and infiltration^[152]^. OX40 (CD134) which is also a TNF receptor expressed on CD4+ and CD8+ T cells is another stimulatory molecule that can serve as a possible target to overcome resistance to anti-PD-1/CTLA-4 antibody therapy^[153]^. Successful research on monotherapy or combination therapy drove more current clinical trials using agnostic anti-OX40 antibody^[154-156]^.

Additional inhibitory checkpoints are T cell immunoglobulin and mucin 3 (Tim-3), T cell immunoglobulin and immunoreceptor tyrosine-based inhibitory motif (ITIM) Domain (TIGIT), lymphocyte-activation gene-3 (LAG-3), and V-domain Ig suppressor of T cell activation (VISTA)^[157]^. Tim-3 is usually expressed on T cells, macrophages, and NK-cells. Tim-3 and PD-1 are expressed on T cells simultaneously and their co-expression is associated with poor prognosis in several types of cancer such as melanoma, NSCLC, and renal cell carcinoma^[158-160]^. A phase I study of anti-Tim-3 monoclonal antibody is ongoing (NCT02817633). TIGIT is another inhibitory receptor mainly expressed on T cells and NK cells. With its ligand, poliovirus receptor, the role of TIGIT in the tumor immune environment resembles the PD-1/PD-L1 interaction in tumor immunosuppression^[161,162]^. An early-phase clinical trial mainly targeting hematological malignancies is currently ongoing (NCT04045028), possibly allowing new treatment strategies and improving clinical responses^[163]^.

LAG-3 is found on T cells, NK cells, B cells, and DCs. There are mainly three ligands for LAG3, MHC class II molecules on APCs, Galectin-3, and liver sinusoidal endothelial cell lectin (LSECtin)^[164,165]^. Their roles are competitive inhibition of antigen and T cell receptor, suppression of tumor-specific T cell responses, and the inhibition of antitumor T cell responses, respectively. Although LAG-3 seems to have limited functionality compared to other inhibitory checkpoints, monotherapy or combination therapy using an anti-LAG3 antibody has been studied, showing some efficacy^[166,167]^.

#### Indoleamine 2,3-dioxygenase

Indoleamine 2,3-dioxygenase (IDO) is an enzyme that has a role in tryptophan catabolism to kynurenine. It is known to inhibit effector T cell functions, contributing to peripheral tolerance. IFN-gamma induces IDO expression, which is associated with a poor prognosis in many types of cancers^[168,169]^. IDO is involved in immune escape by inhibiting effector T cells and with neovascularization by modulating the level of inflammatory cytokines such as IFN-gamma and IL-6, leading to tumor cell proliferation^[170]^. For these reasons, IDO gained interest as a targetable molecule in the era of immunotherapy and several early-phase trials using IDO inhibitors are currently underway. The use of the IDO-1 inhibitor, epacadostat, as monotherapy was evaluated in a phase I trial, showing tolerability but a limited objective response, presumably due to other pathways resulting in immune escape^[171]^. To suppress these immune escape mechanisms, combination therapies using IDO inhibitors with other agents such as PD-1 inhibitors and chemotherapy have been evaluated. Several early-phase trials combining the IDO inhibitors epacadostat and indoximod with a PD-1 inhibitor have showed encouraging results in solid tumor patients. However, the phase III KEYNOTE-252/ECHO-301 trial, which tested epacadostat in combination with pembrolizumab in advanced melanoma patients unexpectedly, had negative results^[172,173]^. Chemotherapy or radiation therapy, which induces antigen presentation from cell death are possibly needed to establish robust strategies for using an IDO inhibitor. Thus, triple therapy trials using chemotherapy, vaccine, or other checkpoint inhibitors in addition to IDO inhibitor and PD-1 inhibitor are ongoing (NCT03322566) (NCT03661320) (NCT03519256) (NCT03006302) (NCT02658890).

#### Angiogenesis

Angiogenesis is known to play an essential role in tumor growth and metastasis in solid tumors^[174]^. Anti-angiogenetic agents that inhibit VEGF or its receptor VEGFR have been approved for several malignancies such as colon and ovarian cancer. However, VEGF not only has the ability to enhance angiogenesis but also has an immunosuppressive role by increasing Fas ligand expression in endothelial cells to reduce effector T cells and upregulate Tregs in the TME, contributing to ICI therapy resistance^[175-177]^. For this reason, targeting VEGF is an important strategy to reinforce anti-tumor immunity^[178-182]^. With this in mind, clinical trials testing the efficacy of combination therapy using an angiogenesis inhibitor and ICI are being conducted and have demonstrated positive results in some phase 3 trials^[183-185]^.

#### Enteric microbiome

The enteric microbiome is associated with a wide variety of diseases including inflammatory bowel disease, arthritis, and Parkinson’s disease^[186]^. It is now understood that the microbiome can have an influence on cancer prognosis^[187]^. Several studies have elucidated the relationship between the enteric microbiome and cancer growth over the last few years. The gut microbiota may modulate immunity against tumor cells through the activation of DCs in the intestine. Moreover, the migration of enteric bacteria to the TME along with the activation of effector T cells also has an important role to invoke anti-cancer immunity^[188]^. Products from enterobacterial flora such as pathogen-associated molecular patterns could induce antigen presentation and result in the activation of effector T cells, DCs, macrophages, and NK cells^[188,189]^. In concordance with these results, recent data from several groups have shown a correlation between the gut microbiome, antibiotics use, and the efficacy of immunotherapy^[190]^. While species like *Bifidobacterium, Collinsella, Faecalibacterium, Ruminococcaceae*, *Akkermansia*, and *Enterococcus* were found to be enriched in responders to anti-PD-1 therapy, species such as *Bacteroides* were enriched in non-responders^[190-192]^. Also, *Bacteroides* species were associated with positive efficacy from the anti-CTLA-4 antibody. Fecal microbial transplantation from patients with good response to anti-CTLA-4 antibody and supplementation of *Bacteroides* species to germ-free mice resulted in improved response to anti-CTLA-4 antibody^[193]^. Additionally, solid tumor patients such as non-small cell lung cancer who received antibiotics just before the start of anti-PD-1 antibody had lower response rates and poorer survival^[194,195]^. As a result, modification of the enteric microbiome seems to augment the clinical efficacy of ICI therapy. Previous studies using mouse models have found that probiotics or fecal microbiota transplantation combined with ICIs induces a synergetic effect and evasion from treatment resistance. For example, oral administration of *Bifidobacterium* with anti-PD-L1 antibody therapy was effective in diminishing tumor growth through the augmented function of DCs leading to CD8+ T cell priming and accumulation in the TME^[196]^. At this time, some enteric species seem beneficial for reinforcing the efficacy of ICIs but the precise immunological and metabolic effects of microbiome-based interventions on cancer immunotherapy remain unknown. However, the enteric microbiome clearly affects cancer immunity and these findings have set the stage for exciting clinical trials in this arena^[197]^.

## Conclusion

Our comprehensive literature review has uncovered a wide range of immunotherapy resistance mechanisms and potential areas of further research into therapeutic strategies involving genetics, to the cellular microenvironment and the microbiota. Due to the accumulation of evidence supporting the promising results of combination therapy, including checkpoint inhibitors and other agents, systemically and locally [Tables 1 and 2], it is vital to optimize treatment based on the mechanism of the patient’s relapse or recurrence. More importantly, future discoveries should include drug resistance biomarker development to improve decision making for each patient. This emphasizes the importance of well-designed clinical trials, which may further enhance our understanding of immunotherapy resistance and strategies to overcome them.

**Table 1:** Systemic therapies with the potential to overcome ICI resistance and their putative mechanisms

Systemic treatment	Representative agents	Mechanism of overcoming resistance	Ref.
Immune checkpoint inhibitor	Anti-PD-1 inhibitor	Activates effector T cells Reactivates antigen-T cell connection Increases T cell proliferation Reverses T cell exhaustion Decreases Tregs	[10-13]
	Anti-PD-L1 inhibitor		[10-13]
	Anti-CTLA-4 inhibitor		[8,9,14]
	Anti-TIGIT inhibitor		[161-163]
	Anti-Tim-3 inhibitor		[158-160]
	Anti-LAG-3 inhibitor		[166,167]
	Anti-VISTA inhibitor		[199,200]
	CA-170	Dual blockade of PD-1/PD-L1 and VISTA	[201]
Immune-stimulatory molecule agonist	CD40 agonist	Upregulates T cell infiltration and activation Increases DCs infiltration, activates APC maturation, decreases MDSCs	[151,152]
	OX40 agonist	Suppresses Tregs Increases TIL	[152-156]
	41BB agonist	Activates effector T cells	[202]
	ICOS agonist	Helps T cell memory formation	[203]
	GITR agonist	Helps T cell memory formation	[204]
Chemotherapy	Gemcitabine	Excises MDSCs	[205]
	Temozolomide	Increases mutations and neoantigens	[206]
	Cisplatin	Increases CD8+ T cells and PD-L1 expression	[207]
	Paclitaxel	Activates immunogenic cell damage	[208]
Molecular targeting therapy			
MAPK pathway	EGFR inhibitor	Promotes CD8+ T cell activation and infiltration within the TME Induces the expression of tumor antigens	[61-63]
	MEK inhibitor		
	BRAF inhibitor		
PI3K-AKT pathway	AKT inhibitor	Decreases the production of immunosuppressive cytokines	[65]
	mTOR inhibitor		
WNT/β-catenin pathway	β-catenin inhibitor	Increases T cell-mediated cytotoxicity	[209]
	STAT3 inhibitor	Suppresses WNT/β-catenin pathway	[210]
IFN-JAK-STAT pathway	JAK2 inhibitor	Downregulates oncogenic STAT signaling Preserves inflammatory signals for anti-tumor immunity	[211]
EPHA2-TGF-β-PTSG2 pathway	TGF-β inhibitor	Activates T cell infiltration Decreases fibrosis around tumor cells Decreases Tregs and EMT	[137,140-143]
	CDK4/6 inhibitor	Suppresses immune evasion Increases antigen presentation	[212]
	SERPINB9 inhibitor	Downregulates immune evasion	[213]
Immunomodulating molecules	IDO inhibitor	Activates effector T cells Inhibits neovascularization Decreases inflammatory cytokines	[172,173]
Adenosine axis	Anti-CD38 inhibitor	Increases effector T cells Decreases MDSCs and Tregs	[214,215]
	Anti-CD39 inhibitor	Increases effector T cells, IFNγ, and DCs	[216,217]
	Anti-CD73 inhibitor	Increases CD4+ T cells and IFNγ	[218]
	A2R antagonist	Activates effector T cells	[219]
Angiogenesis	Anti-VEGF/VEGFR inhibitor	Inhibits angiogenesis Upregulates proinflammatory cytokines Increases antigen-specific T cell migration	[177-181,183-185]
Targeting immunosuppressive molecules			
Tregs	Anti-CCR-4 inhibitor	Impairs Tregs	[220]
	Fc-region-modified anti-CTLA-4 antibody	Depletes intratumoral Tregs	[95,96]
MDSCs	PI3Kδ/γ inhibitor	Activates CD8+ T cells	[105]
	Agonistic TRAIL-R antibody	Activates apoptosis	[106]
	ATRA	Decreases circulating MDSCs	[108-110]
Tumor-associated macrophages (TAMs)	CXCR-4 inhibitor	Inhibits TAMs	[114]
	CSF-1R inhibitor	Inhibits TAMs	[115,116]
	MARCO inhibitor	Inhibits TAMs	[116]
	PD-1/PD-L1 inhibitor	Inhibits PD-1 on TAMs leading to macrophage phagocytosis	[117]
AXL	AXL inhibitor	Inhibits EMT and T cell exclusion	[144,145]
Cytokines	IL-2	Enhances CD8+ T cell response	[221]
	IL-12	Activates effector T cells and NK cells Increases IFNγ	[222,223]
	IL-15	Expands NK cells	[224]
Chemokine inhibition	CCR-1 antagonist	Reduces MDSCs in the TME	[225]
	CCR-2 antagonist	Reduces MDSCs in the TME	[226,227]
	Anti-IL-8 antibody	Inhibits mesenchymal proteins Enhances NK and T cell-mediated killing Reduces MDSCs	[228]
	CXCR-1/2 inhibitor	Reduces MDSCs Inhibits EMT	[229]
	CXCR-2 inhibitor	Suppresses neutrophil/myeloid-derived suppressor cells Improves T cell entry to the TME	[230]
Adoptive cell transfer	TIL ACT	Increases T cell infiltration	[231]
	CART	Induces attack by T cells with tumor antigen-specific engineered TCR	[232]
Cancer vaccine	Long peptide vaccine	Presents neoantigens to APCs	[233]
	RNA vaccine	Induces neoantigen production	[233]
	DC vaccine	Ameliorates antigen presentation Activates CD8+ effector T cells	[233]
	Vaccine targeting brachyury	Increases CD8+ T cells Decreases EMT	[146-149]
Epigenetic modulators	DNA methyltransferase inhibitor	Improves antigen processing and presentation Improves chemokine function Reverses T cell exhaustion	[43-45]
	Histone deacetylase (HDAC) inhibitor	Suppresses immunosuppressive cells in the TME	[46-52]
STING pathway activator	CDG, CDN STING agonist ADCs Modified STING agonist	Activates APCs, CD8+ T cells, and DCs Enhances STING pathway	[73-81]
Gut microbiome modification	Probiotics	Decreases immune-related adverse effects Increases intratumoral DCs and T cell priming	[196,234]
	Fecal transplantation	Increases intratumoral DCs and effector T cells Decreases CD4+ FoxP3+ Tregs in the TME	[193]
	Use or avoid antibiotics	Controversial	[194,195]
Other immune-related targets	Exosomal PD-L1 inhibitor	Activates T cells in the draining lymph node	[85]

ICI: Immune checkpoint inhibitor; PD-1: programmed death 1; PD-L1: programmed cell death ligand 1; MDSC: myeloid-derived suppressor cell; IDO: Indoleamine 2,3-dioxygenase; APC: antigen-presenting cell; TME: tumor microenvironment; DCs: dendritic cells; CDG: cyclic diguanylate; CDN: cyclic dinucleotide; EMT: epithelial-mesenchymal transition; VEGF: vascular endothelial growth factor

**Table 2:** Loco-regional strategies to overcome ICI resistance and putative mechanisms through which this occurs

Local treatment	Agents	Mechanism	Ref.
Ablation			
Radiotherapy	External irradiation	Local control Increases antigen presentation	[235-237]
	RFA	Local control Increases antigen presentation	[238]
Incision	-	Local control Modifies heterogeneity	
Cryoablation	-	Local control Increases antigen presentation	[239]
Intralesional administration			
Immune checkpoint inhibitor	Anti-CTLA-4 inhibitor	Increases CD8+ T cells in the TME	[240]
Cytokine	IL-2	Induces IFNγ and CD8+ T cells	[240-243]
Bacteria	Engineered bacteria	Activates TIL, leading to systemic antitumor immunity	[244]
Oncolytic virus	GM-CSF encoding HSV-1	Enhances CD8+ T cell infiltration	[245]
	Coxsackie A virus	Enhances CD8+ T cell infiltration	[245]
Targeting Toll-like receptor	TLR agonist	Increases type I IFN and CD8+ T cell infiltration Depletes Tregs	[97,245]
Vaccine	BCG vaccine	Induces TILs Suppresses MDSCs	[246]

ICI: Immune checkpoint inhibitor; CTLA-4: cytotoxic T lymphocyte-associated protein 4; TME: tumor microenvironment; IFN: interferon; IL: interleukin

The immuno-oncology therapeutic landscape has undergone significant changes over the last decade due to the emergence of ICIs. However, many patients still do not respond to these agents due to primary immunotherapy resistance. Furthermore, there are patients who despite responding initially, develop resistance later. One approach to reduce the number of response failures will be to identify biomarkers predictive of response that may then allow better screening of patients before undergoing ICI treatment and then, offer alternative therapeutics as indicated. The other approach will be to identify the mechanisms of primary or acquired resistance and to find new combination therapies to overcome these issues [Figure 2B]. Due to the limited number of animal models that can be used to generate pre-clinical data on these immune-oncology agents, it is imperative to maximize the utility of preclinical studies focusing on mechanisms of action and to quickly pivot over to clinical trials to test their safety and efficacy. Moreover, it is key to further support the preclinical hypothesis with patient biopsy specimens and peripheral blood samples under clinical trial settings, which will then become the most important correlative research studies in order to advance the development of immune-oncology agents.

Previously, correlative research studies have been dependent on an individual researcher’s laboratory assays. This approach poses a significant bottleneck however, when molecular or immunological analysis is done on patient tissues across multiple clinical studies since typically, the assay used by one investigator cannot be validated at the pre-analytical or analytical stage, and hence data comparison and analysis become difficult. To address this most critical issue, the NCI Division of Cancer Treatment and Diagnosis (DCTD) and Cancer Therapy Evaluation Program (CTEP) have established a “Cancer Immune Monitoring and Analysis Centers (CIMACs) and its associated database center “Cancer Immunological Data Commons” (CIDC) using the NIH U24 grant mechanism^[198]^. The Investigational Drug Branch (IDB) at CTEP supports early phase 1-2 trials under their network, Experimental Therapeutics Clinical Trials Network and these network investigators need a laboratory resource with biomarker expertise with which they can be closely aligned with. Therefore, immunotherapy trials and correlative scientific assays can be developed jointly. Once the trial is completed, trial results are submitted to the CIDC for secondary analyses by the greater research community.

The NCI DCTD has another resource named the “Drug Resistance and Sensitivity” (DRSC) program, which was initiated to support the research on the mechanisms of action of drug resistance and sensitivity. However, the uniqueness of this approach is not to focus on preclinical models, which are commonly addressed within regular RO1 type of grants but to develop the hypothesis-driven research using patient-derived samples from DCTD sponsored trials. Thus, the U54 DRSC and CIMACs can complement each other in research aimed at immune-oncology agent drug resistance mechanisms under clinical trials. Data generated from the DRSC network activities will then be deposited to the NCI Genomic Data Commons, allowing further analysis by the extramural research community.

The advent of immunotherapy has paved the way to a very exciting time for the treatment of many different cancers. As our understanding of these immune agents and the different ways of treatment resistance develops, it is more important than ever to build upon our knowledge of these mechanisms to help identify avenues to overcome it and thereby, increase the number of patients who can benefit from these novel therapies.

## Declarations

### Copyright

© The Author(s) 2020.

## References

[1] Sharma P, Hu-Lieskovan S, Wargo JA, Ribas A (2017). Primary, Adaptive, and acquired resistance to cancer immunotherapy.. Cell.

[2] Wei SC, Duffy CR, Allison JP (2018). Fundamental mechanisms of immune checkpoint blockade therapy.. Cancer Discov.

[3] Coley WB (1891). Contribution to the Knowledge of Sarcoma.. Ann Surg.

[4] Hoption Cann SA, van Netten JP, van Netten C (2003). Dr William coley and tumour regression: a place in history or in the future.. Postgrad Med J.

[5] Ribas A, Wolchok JD (2018). Cancer immunotherapy using checkpoint blockade.. Science.

[6] Fares CM, Van Allen EM, Drake CG, Allison JP, Hu-Lieskovan S (2019). Mechanisms of resistance to immune checkpoint blockade: why does checkpoint inhibitor immunotherapy not work for all patients?. Am Soc Clin Oncol Educ Book.

[7] De Sousa Linhares A, Leitner J, Grabmeier-Pfistershammer K, Steinberger P (2018). Not all immune checkpoints are created equal.. Front Immunol.

[8] Brunet JF, Denizot F, Luciani MF, Roux-Dosseto M, Suzan M (1987). A new member of the immunoglobulin superfamily--CTLA-4.. Nature.

[9] Krummel MF, Allison JP (1995). CD28 and CTLA-4 have opposing effects on the response of T cells to stimulation.. J Exp Med.

[10] Ishida Y, Agata Y, Shibahara K, Honjo T (1992). Induced expression of PD-1, a novel member of the immunoglobulin gene superfamily, upon programmed cell death.. EMBO J.

[11] Iwai Y, Ishida M, Tanaka Y, Okazaki T, Honjo T (2002). Involvement of PD-L1 on tumor cells in the escape from host immune system and tumor immunotherapy by PD-L1 blockade.. Proc Natl Acad Sci U S A.

[12] Strome SE, Dong H, Tamura H, Voss SG, Flies DB (2003). B7-H1 blockade augments adoptive T-cell immunotherapy for squamous cell carcinoma.. Cancer Res.

[13] Hirano F, Kaneko K, Tamura H, Dong H, Wang S (2005). Blockade of B7-H1 and PD-1 by monoclonal antibodies potentiates cancer therapeutic immunity.. Cancer Res.

[14] Lipson EJ, Drake CG (2011). Ipilimumab: an anti-CTLA-4 antibody for metastatic melanoma.. Clin Cancer Res.

[15] Sambi M, Bagheri L, Szewczuk MR (2019). Current challenges in cancer immunotherapy: multimodal approaches to improve efficacy and patient response rates.. J Oncol.

[16] Syn NL, Teng MWL, Mok TSK, Soo RA (2017). De-novo and acquired resistance to immune checkpoint targeting.. Lancet Oncol.

[17] Jenkins RW, Barbie DA, Flaherty KT (2018). Mechanisms of resistance to immune checkpoint inhibitors.. Br J Cancer.

[18] Gide TN, Wilmott JS, Scolyer RA, Long GV (2018). Primary and acquired resistance to immune checkpoint inhibitors in metastatic melanoma.. Clin Cancer Res.

[19] Gubin MM, Zhang X, Schuster H, Caron E, Ward JP (2014). Checkpoint blockade cancer immunotherapy targets tumour-specific mutant antigens.. Nature.

[20] Schumacher TN, Schreiber RD (2015). Neoantigens in cancer immunotherapy.. Science.

[21] Nowicki TS, Hu-Lieskovan S, Ribas A (2018). Mechanisms of resistance to PD-1 and PD-L1 blockade.. Cancer J.

[22] Rizvi NA, Hellmann MD, Snyder A, Kvistborg P, Makarov V (2015). Cancer immunology. Mutational landscape determines sensitivity to PD-1 blockade in non-small cell lung cancer.. Science.

[23] van Rooij N, van Buuren MM, Philips D, Velds A, Toebes M (2013). Tumor exome analysis reveals neoantigen-specific T-cell reactivity in an ipilimumab-responsive melanoma.. J Clin Oncol.

[24] Le DT, Durham JN, Smith KN, Wang H, Bartlett BR (2017). Mismatch repair deficiency predicts response of solid tumors to PD-1 blockade.. Science.

[25] de Velasco G, Miao D, Voss MH, Hakimi AA, Hsieh JJ (2016). Tumor mutational load and immune parameters across metastatic renal cell carcinoma risk groups.. Cancer Immunol Res.

[26] Llosa NJ, Cruise M, Tam A, Wicks EC, Hechenbleikner EM (2015). The vigorous immune microenvironment of microsatellite instable colon cancer is balanced by multiple counter-inhibitory checkpoints.. Cancer Discov.

[27] Neefjes J, Jongsma ML, Paul P, Bakke O (2011). Towards a systems understanding of MHC class I and MHC class II antigen presentation.. Nat Rev Immunol.

[28] Marincola FM, Jaffee EM, Hicklin DJ, Ferrone S (2000). Escape of human solid tumors from T-cell recognition: molecular mechanisms and functional significance.. Adv Immunol.

[29] Sucker A, Zhao F, Real B, Heeke C, Bielefeld N (2014). Genetic evolution of T-cell resistance in the course of melanoma progression.. Clin Cancer Res.

[30] Rooney MS, Shukla SA, Wu CJ, Getz G, Hacohen N (2015). Molecular and genetic properties of tumors associated with local immune cytolytic activity.. Cell.

[31] Zhao F, Sucker A, Horn S, Heeke C, Bielefeld N (2016). Melanoma lesions independently acquire T-cell resistance during metastatic latency.. Cancer Res.

[32] Zaretsky JM, Garcia-Diaz A, Shin DS, Escuin-Ordinas H, Hugo W (2016). Mutations associated with acquired resistance to PD-1 blockade in melanoma.. N Engl J Med.

[33] Sade-Feldman M, Jiao YJ, Chen JH, Rooney MS, Barzily-Rokni M (2017). Resistance to checkpoint blockade therapy through inactivation of antigen presentation.. Nat Commun.

[34] Pereira C, Gimenez-Xavier P, Pros E, Pajares MJ, Moro M (2017). Genomic profiling of patient-derived xenografts for lung cancer identifies B2M inactivation impairing immunorecognition.. Clin Cancer Res.

[35] Le DT, Uram JN, Wang H, Bartlett BR, Kemberling H (2015). PD-1 Blockade in Tumors with Mismatch-Repair Deficiency.. N Engl J Med.

[36] Dudley JC, Lin MT, Le DT, Eshleman JR (2016). Microsatellite Instability as a Biomarker for PD-1 Blockade.. Clin Cancer Res.

[37] Karpf AR, Jones DA (2002). Reactivating the expression of methylation silenced genes in human cancer.. Oncogene.

[38] Kim HJ, Bae SC (2011). Histone deacetylase inhibitors: molecular mechanisms of action and clinical trials as anti-cancer drugs.. Am J Transl Res.

[39] Yoshihama S, Roszik J, Downs I, Meissner TB, Vijayan S (2016). NLRC5/MHC class I transactivator is a target for immune evasion in cancer.. Proc Natl Acad Sci U S A.

[40] Burr ML, Sparbier CE, Chan KL, Chan YC, Kersbergen A (2019). An Evolutionarily Conserved Function of Polycomb Silences the MHC Class I Antigen Presentation Pathway and Enables Immune Evasion in Cancer.. Cancer Cell.

[41] Dunn GP, Sheehan KC, Old LJ, Schreiber RD (2005). IFN unresponsiveness in LNCaP cells due to the lack of JAK1 gene expression.. Cancer Res.

[42] Heninger E, Krueger TE, Lang JM (2015). Augmenting antitumor immune responses with epigenetic modifying agents.. Front Immunol.

[43] Li X, Zhang Y, Chen M, Mei Q, Liu Y (2017). Increased IFNγ^+^ T cells are responsible for the clinical responses of low-dose DNA-demethylating agent decitabine antitumor therapy.. Clin Cancer Res.

[44] Ghoneim HE, Fan Y, Moustaki A, Abdelsamed HA, Dash P (2017). De Novo epigenetic programs inhibit PD-1 blockade-mediated T cell rejuvenation.. Cell.

[45] Terracina KP, Graham LJ, Payne KK, Manjili MH, Baek A (2016). DNA methyltransferase inhibition increases efficacy of adoptive cellular immunotherapy of murine breast cancer.. Cancer Immunol Immunother.

[46] Shen L, Ciesielski M, Ramakrishnan S, Miles KM, Ellis L (2012). Class I histone deacetylase inhibitor entinostat suppresses regulatory T cells and enhances immunotherapies in renal and prostate cancer models.. PLoS One.

[47] Orillion A, Hashimoto A, Damayanti N, Shen L, Adelaiye-Ogala R (2017). Entinostat neutralizes myeloid-derived suppressor cells and enhances the antitumor effect of PD-1 inhibition in murine models of lung and renal cell carcinoma.. Clin Cancer Res.

[48] Christmas BJ, Rafie CI, Hopkins AC, Scott BA, Ma HS (2018). Entinostat converts immune-resistant breast and pancreatic cancers into checkpoint-responsive tumors by reprogramming tumor-infiltrating MDSCs.. Cancer Immunol Res.

[49] Agarwala SS, Moschos SJ, Johnson ML, Opyrchal M, Gabrilovich D (2018). Efficacy and safety of entinostat (ENT) and pembrolizumab (PEMBRO) in patients with melanoma progressing on or after a PD-1/L1 blocking antibody.. J Clin Oncol.

[50] Sullivan RJ, Moschos SJ, Johnson ML, Opyrchal M, Ordentlich P (2019). Abstract CT072: efficacy and safety of entinostat (ENT) and pembrolizumab (PEMBRO) in patients with melanoma previously treated with anti-PD1 therapy.. Cancer Res.

[51] Hellmann M, Jänne P, Opyrchal M, Hafez N, Raez L (2018). Efficacy/Safety of Entinostat (ENT) and Pembrolizumab (PEMBRO) in NSCLC patients previously treated with anti-PD-(L)1 therapy.. J Thorac Oncol.

[52] Gray JE, Saltos A, Tanvetyanon T, Haura EB, Creelan B (2019). Phase I/Ib study of pembrolizumab plus vorinostat in advanced/metastatic non-small cell lung cancer.. Clin Cancer Res.

[53] Kalbasi A, Ribas A (2020). Tumour-intrinsic resistance to immune checkpoint blockade.. Nat Rev Immunol.

[54] Spranger S, Bao R, Gajewski TF (2015). Melanoma-intrinsic β-catenin signalling prevents anti-tumour immunity.. Nature.

[55] Molina JR, Adjei AA (2006). The Ras/Raf/MAPK pathway.. J Thorac Oncol.

[56] Zhao X, Subramanian S (2018). Oncogenic pathways that affect antitumor immune response and immune checkpoint blockade therapy.. Pharmacol Ther.

[57] Liu C, Peng W, Xu C, Lou Y, Zhang M (2013). BRAF inhibition increases tumor infiltration by T cells and enhances the antitumor activity of adoptive immunotherapy in mice.. Clin Cancer Res.

[58] Chen N, Fang W, Zhan J, Hong S, Tang Y (2015). Upregulation of PD-L1 by EGFR activation mediates the immune escape in EGFR-driven NSCLC: implication for optional immune targeted therapy for NSCLC patients with EGFR mutation.. J Thorac Oncol.

[59] Ota K, Azuma K, Kawahara A, Hattori S, Iwama E (2015). Induction of PD-L1 expression by the EML4-ALK oncoprotein and downstream signaling pathways in non-small cell lung cancer.. Clin Cancer Res.

[60] Sumimoto H, Takano A, Teramoto K, Daigo Y (2016). RAS-mitogen-activated protein kinase signal is required for enhanced PD-L1 expression in human lung cancers.. PLoS One.

[61] Donia M, Fagone P, Nicoletti F, Andersen RS, Hogdall E (2012). BRAF inhibition improves tumor recognition by the immune system: potential implications for combinatorial therapies against melanoma involving adoptive T-cell transfer.. Oncoimmunology.

[62] Loi S, Dushyanthen S, Beavis PA, Salgado R, Denkert C (2016). RAS/MAPK activation is associated with reduced tumor-infiltrating lymphocytes in triple-negative breast cancer: therapeutic cooperation between MEK and PD-1/PD-L1 immune checkpoint inhibitors.. Clin Cancer Res.

[63] Liu L, Mayes PA, Eastman S, Shi H, Yadavilli S (2015). The BRAF and MEK inhibitors dabrafenib and trametinib: effects on immune function and in combination with immunomodulatory antibodies targeting PD-1, PD-L1, and CTLA-4.. Clin Cancer Res.

[64] Cantley LC (2002). The phosphoinositide 3-kinase pathway.. Science.

[65] Peng W, McKenzie JA, Hwu P (2016). Complementing T-cell function: an inhibitory role of the complement system in T-cell-mediated antitumor immunity.. Cancer Discov.

[66] George S, Miao D, Demetri GD, Adeegbe D, Rodig SJ (2017). Loss of PTEN is associated with resistance to anti-PD-1 checkpoint blockade therapy in metastatic uterine leiomyosarcoma.. Immunity.

[67] Zhan T, Rindtorff N, Boutros M (2017). Wnt signaling in cancer.. Oncogene.

[68] Luke JJ, Bao R, Sweis RF, Spranger S, Gajewski TF (2019). WNT/beta-catenin pathway activation correlates with immune exclusion across human cancers.. Clin Cancer Res.

[69] Benci JL, Xu B, Qiu Y, Wu TJ, Dada H (2016). Tumor interferon signaling regulates a multigenic resistance program to immune checkpoint blockade.. Cell.

[70] Gao J, Shi LZ, Zhao H, Chen J, Xiong L (2016). Loss of IFN-gamma pathway genes in tumor cells as a mechanism of resistance to Anti-CTLA-4 therapy.. Cell.

[71] Shin DS, Zaretsky JM, Escuin-Ordinas H, Garcia-Diaz A, Hu-Lieskovan S (2017). Primary resistance to PD-1 blockade mediated by JAK1/2 mutations.. Cancer Discov.

[72] Woo SR, Fuertes MB, Corrales L, Spranger S, Furdyna MJ (2014). STING-dependent cytosolic DNA sensing mediates innate immune recognition of immunogenic tumors.. Immunity.

[73] Corrales L, Gajewski TF (2015). Molecular pathways: targeting the stimulator of interferon genes (STING) in the immunotherapy of cancer.. Clin Cancer Res.

[74] Corrales L, Glickman LH, McWhirter SM, Kanne DB, Sivick KE (2015). Direct activation of STING in the tumor microenvironment leads to potent and systemic tumor regression and immunity.. Cell Rep.

[75] Fu J, Kanne DB, Leong M, Glickman LH, McWhirter SM (2015). STING agonist formulated cancer vaccines can cure established tumors resistant to PD-1 blockade.. Sci Transl Med.

[76] Ager CR, Reilley MJ, Nicholas C, Bartkowiak T, Jaiswal AR (2017). Intratumoral STING activation with T-cell checkpoint modulation generates systemic antitumor immunity.. Cancer Immunol Res.

[77] Rivera Vargas T, Benoit-Lizon I, Apetoh L (2017). Rationale for stimulator of interferon genes-targeted cancer immunotherapy.. Eur J Cancer.

[78] Ohkuri T, Ghosh A, Kosaka A, Zhu J, Ikeura M (2014). STING contributes to antiglioma immunity via triggering type I IFN signals in the tumor microenvironment.. Cancer Immunol Res.

[79] Koshy ST, Cheung AS, Gu L, Graveline AR, Mooney DJ (2017). Liposomal delivery enhances immune activation by STING agonists for cancer immunotherapy.. Adv Biosyst.

[80] Wilson DR, Sen R, Sunshine JC, Pardoll DM, Green JJ (2018). Biodegradable STING agonist nanoparticles for enhanced cancer immunotherapy.. Nanomedicine.

[81] Challa SV, Zhou S, Sheri A, Padmanabhan S, Meher G (2017). Preclinical studies of SB 11285, a novel STING agonist for immuno-oncology.. J Clin Oncol.

[82] Ricklefs FL, Alayo Q, Krenzlin H, Mahmoud AB, Speranza MC (2018). Immune evasion mediated by PD-L1 on glioblastoma-derived extracellular vesicles.. Sci Adv.

[83] Theodoraki MN, Yerneni SS, Hoffmann TK, Gooding WE, Whiteside TL (2018). Clinical significance of PD-L1(+) exosomes in plasma of head and neck cancer patients.. Clin Cancer Res.

[84] Chen G, Huang AC, Zhang W, Zhang G, Wu M (2018). Exosomal PD-L1 contributes to immunosuppression and is associated with anti-PD-1 response.. Nature.

[85] Poggio M, Hu T, Pai CC, Chu B, Belair CD (2019). Suppression of exosomal PD-L1 induces systemic anti-tumor immunity and memory.. Cell.

[86] Rudensky AY (2011). Regulatory T cells and Foxp3.. Immunol Rev.

[87] Sakaguchi S, Yamaguchi T, Nomura T, Ono M (2008). Regulatory T cells and immune tolerance.. Cell.

[88] Liston A, Gray DH (2014). Homeostatic control of regulatory T cell diversity.. Nat Rev Immunol.

[89] Setoguchi R, Hori S, Takahashi T, Sakaguchi S (2005). Homeostatic maintenance of natural Foxp3(+) CD25(+) CD4(+) regulatory T cells by interleukin (IL)-2 and induction of autoimmune disease by IL-2 neutralization.. J Exp Med.

[90] Takahashi T, Tagami T, Yamazaki S, Uede T, Shimizu J (2000). Immunologic self-tolerance maintained by CD25(+)CD4(+) regulatory T cells constitutively expressing cytotoxic T lymphocyte-associated antigen 4.. J Exp Med.

[91] Cao X, Cai SF, Fehniger TA, Song J, Collins LI (2007). Granzyme B and perforin are important for regulatory T cell-mediated suppression of tumor clearance.. Immunity.

[92] Fridman WH, Pages F, Sautes-Fridman C, Galon J (2012). The immune contexture in human tumours: impact on clinical outcome.. Nat Rev Cancer.

[93] Hamid O, Schmidt H, Nissan A, Ridolfi L, Aamdal S (2011). A prospective phase II trial exploring the association between tumor microenvironment biomarkers and clinical activity of ipilimumab in advanced melanoma.. J Transl Med.

[94] Simpson TR, Li F, Montalvo-Ortiz W, Sepulveda MA, Bergerhoff K (2013). Fc-dependent depletion of tumor-infiltrating regulatory T cells co-defines the efficacy of anti-CTLA-4 therapy against melanoma.. J Exp Med.

[95] Ha D, Tanaka A, Kibayashi T, Tanemura A, Sugiyama D (2019). Differential control of human Treg and effector T cells in tumor immunity by Fc-engineered anti-CTLA-4 antibody.. Proc Natl Acad Sci U S A.

[96] Arce Vargas F, Furness AJS, Litchfield K, Joshi K, Rosenthal R (2018). Fc effector function contributes to the activity of human anti-CTLA-4 antibodies.. Cancer Cell.

[97] Sharma N, Vacher J, Allison JP (2019). TLR1/2 ligand enhances antitumor efficacy of CTLA-4 blockade by increasing intratumoral Treg depletion.. Proc Natl Acad Sci U S A.

[98] Heath DJ, Vanderkerken K, Cheng X, Gallagher O, Prideaux M (2007). An osteoprotegerin-like peptidomimetic inhibits osteoclastic bone resorption and osteolytic bone disease in myeloma.. Cancer Res.

[99] Bronte V, Wang M, Overwijk WW, Surman DR, Pericle F (1998). Apoptotic death of CD8+ T lymphocytes after immunization: induction of a suppressive population of Mac-1+/Gr-1+ cells.. J Immunol.

[100] Yang L, DeBusk LM, Fukuda K, Fingleton B, Green-Jarvis B (2004). Expansion of myeloid immune suppressor Gr+CD11b+ cells in tumor-bearing host directly promotes tumor angiogenesis.. Cancer Cell.

[101] Meyer C, Cagnon L, Costa-Nunes CM, Baumgaertner P, Montandon N, Leyvraz L, Michielin O, Romano E, Speiser DE (2014). Frequencies of circulating MDSC correlate with clinical outcome of melanoma patients treated with ipilimumab.. Cancer Immunol Immunother.

[102] de Coana YP, Wolodarski M, Poschke I, Yoshimoto Y, Yang Y (2017). Ipilimumab treatment decreases monocytic MDSCs and increases CD8 effector memory T cells in long-term survivors with advanced melanoma.. Oncotarget.

[103] Kaneda MM, Messer KS, Ralainirina N, Li H, Leem CJ (2016). PI3Kgamma is a molecular switch that controls immune suppression.. Nature.

[104] Foubert P, Kaneda MM, Varner JA (2017). PI3Kgamma activates integrin alpha4 and promotes immune suppressive myeloid cell polarization during tumor progression.. Cancer Immunol Res.

[105] Davis RJ, Moore EC, Clavijo PE, Friedman J, Cash H (2017). Anti-PD-L1 efficacy can be enhanced by inhibition of myeloid-derived suppressor cells with a selective inhibitor of PI3Kdelta/gamma.. Cancer Res.

[106] Wiezorek J, Holland P, Graves J (2010). Death receptor agonists as a targeted therapy for cancer.. Clin Cancer Res.

[107] Mirza N, Fishman M, Fricke I, Dunn M, Neuger AM (2006). All-trans-retinoic acid improves differentiation of myeloid cells and immune response in cancer patients.. Cancer Res.

[108] Adamson PC, Reaman G, Finklestein JZ, Feusner J, Berg SL (1997). Phase I trial and pharmacokinetic study of all-trans-retinoic acid administered on an intermittent schedule in combination with interferon-alpha2a in pediatric patients with refractory cancer.. J Clin Oncol.

[109] Kusmartsev S, Cheng F, Yu B, Nefedova Y, Sotomayor E (2003). All-trans-retinoic acid eliminates immature myeloid cells from tumor-bearing mice and improves the effect of vaccination.. Cancer Res.

[110] Tobin RP, Jordan KR, Robinson WA, Davis D, Borges VF (2018). Targeting myeloid-derived suppressor cells using all-trans retinoic acid in melanoma patients treated with Ipilimumab.. Int Immunopharmacol.

[111] Karnes JH, Bastarache L, Shaffer CM, Gaudieri S, Xu Y (2017). Phenome-wide scanning identifies multiple diseases and disease severity phenotypes associated with HLA variants.. Sci Transl Med.

[112] Scala S (2015). Molecular pathways: targeting the CXCR4-CXCL12 axis--untapped potential in the tumor microenvironment.. Clin Cancer Res.

[113] Burger JA, Kipps TJ (2006). CXCR4: a key receptor in the crosstalk between tumor cells and their microenvironment.. Blood.

[114] Hughes R, Qian BZ, Rowan C, Muthana M, Keklikoglou I (2015). Perivascular M2 macrophages stimulate tumor relapse after chemotherapy.. Cancer Res.

[115] Zhu Y, Knolhoff BL, Meyer MA, Nywening TM, West BL (2014). CSF1/CSF1R blockade reprograms tumor-infiltrating macrophages and improves response to T-cell checkpoint immunotherapy in pancreatic cancer models.. Cancer Res.

[116] Mok S, Koya RC, Tsui C, Xu J, Robert L (2014). Inhibition of CSF-1 receptor improves the antitumor efficacy of adoptive cell transfer immunotherapy.. Cancer Res.

[117] Gordon SR, Maute RL, Dulken BW, Hutter G, George BM (2017). PD-1 expression by tumour-associated macrophages inhibits phagocytosis and tumour immunity.. Nature.

[118] Gibney GT, Weiner LM, Atkins MB (2016). Predictive biomarkers for checkpoint inhibitor-based immunotherapy.. The Lancet Oncology.

[119] Tumeh PC, Harview CL, Yearley JH, Shintaku IP, Taylor EJ (2014). PD-1 blockade induces responses by inhibiting adaptive immune resistance.. Nature.

[120] Goff SL, Dudley ME, Citrin DE, Somerville RP, Wunderlich JR (2016). Randomized, prospective evaluation comparing intensity of lymphodepletion before adoptive transfer of tumor-infiltrating lymphocytes for patients with metastatic melanoma.. J Clin Oncol.

[121] Wei F, Zhong S, Ma Z, Kong H, Medvec A (2013). Strength of PD-1 signaling differentially affects T-cell effector functions.. Proc Natl Acad Sci U S A.

[122] Garon EB, Rizvi NA, Hui R, Leighl N, Balmanoukian AS (2015). Pembrolizumab for the treatment of non-small-cell lung cancer.. N Engl J Med.

[123] Patel SP, Kurzrock R (2015). PD-L1 expression as a predictive biomarker in cancer immunotherapy.. Mol Cancer Ther.

[124] Blank CU, Haining WN, Held W, Hogan PG, Kallies A (2019). Defining ‘T cell exhaustion’.. Nat Rev Immunol.

[125] Miller BC, Sen DR, Al Abosy R, Bi K, Virkud YV (2019). Subsets of exhausted CD8(+) T cells differentially mediate tumor control and respond to checkpoint blockade.. Nat Immunol.

[126] Siddiqui I, Schaeuble K, Chennupati V, Fuertes Marraco SA, Calderon-Copete S (2019). Intratumoral Tcf1(+)PD-1(+)CD8(+) T cells with stem-like properties promote tumor control in response to vaccination and checkpoint blockade immunotherapy.. Immunity.

[127] Khan O, Giles JR, McDonald S, Manne S, Ngiow SF (2019). TOX transcriptionally and epigenetically programs CD8(+) T cell exhaustion.. Nature.

[128] Ribas A, Shin DS, Zaretsky J, Frederiksen J, Cornish A (2016). PD-1 blockade expands intratumoral memory t cells.. Cancer Immunol Res.

[129] Horn LA, Fousek K, Palena C (2020). Tumor plasticity and resistance to immunotherapy.. Trends Cancer.

[130] Zheng X, Carstens JL, Kim J, Scheible M, Kaye J (2015). Epithelial-to-mesenchymal transition is dispensable for metastasis but induces chemoresistance in pancreatic cancer.. Nature.

[131] Fischer KR, Durrans A, Lee S, Sheng J, Li F (2015). Epithelial-to-mesenchymal transition is not required for lung metastasis but contributes to chemoresistance.. Nature.

[132] Byers LA, Diao L, Wang J, Saintigny P, Girard L (2013). An epithelial-mesenchymal transition gene signature predicts resistance to EGFR and PI3K inhibitors and identifies Axl as a therapeutic target for overcoming EGFR inhibitor resistance.. Clin Cancer Res.

[133] Waugh DJ, Wilson C (2008). The interleukin-8 pathway in cancer.. Clin Cancer Res.

[134] Cheng Y, Ma XL, Wei YQ, Wei XW (2019). Potential roles and targeted therapy of the CXCLs/CXCR2 axis in cancer and inflammatory diseases.. Biochim Biophys Acta Rev Cancer.

[135] Gregory PA, Bracken CP, Smith E, Bert AG, Wright JA (2011). An autocrine TGF-beta/ZEB/miR-200 signaling network regulates establishment and maintenance of epithelial-mesenchymal transition.. Mol Biol Cell.

[136] Mariathasan S, Turley SJ, Nickles D, Castiglioni A, Yuen K (2018). TGFbeta attenuates tumour response to PD-L1 blockade by contributing to exclusion of T cells.. Nature.

[137] Tauriello DVF, Palomo-Ponce S, Stork D, Berenguer-Llergo A, Badia-Ramentol J (2018). TGFbeta drives immune evasion in genetically reconstituted colon cancer metastasis.. Nature.

[138] Massague J (2012). TGFbeta signalling in context.. Nat Rev Mol Cell Biol.

[139] Pickup M, Novitskiy S, Moses HL (2013). The roles of TGFbeta in the tumour microenvironment.. Nat Rev Cancer.

[140] Dodagatta-Marri E, Meyer DS, Reeves MQ, Paniagua R, To MD (2019). α-PD-1 therapy elevates Treg/Th balance and increases tumor cell pSmad3 that are both targeted by α-TGFβ antibody to promote durable rejection and immunity in squamous cell carcinomas.. J Immunother Cancer.

[141] Knudson KM, Hicks KC, Luo X, Chen JQ, Schlom J (2018). M7824, a novel bifunctional anti-PD-L1/TGFβ Trap fusion protein, promotes anti-tumor efficacy as monotherapy and in combination with vaccine.. Oncoimmunology.

[142] Lan Y, Zhang D, Xu C, Hance KW, Marelli B (2018). Enhanced preclinical antitumor activity of M7824, a bifunctional fusion protein simultaneously targeting PD-L1 and TGF-β.. Sci Transl Med.

[143] Strauss J, Heery CR, Schlom J, Madan RA, Cao L (2018). Phase I trial of M7824 (MSB0011359C), a bifunctional fusion protein targeting PD-L1 and TGFβ, in advanced solid tumors.. Clin Cancer Res.

[144] Paolino M, Penninger JM (2016). The role of TAM family receptors in immune cell function: implications for cancer therapy.. Cancers (Basel).

[145] Aguilera TA, Giaccia AJ (2017). Molecular Pathways: Oncologic pathways and their role in T-cell exclusion and immune evasion-a new role for the AXL receptor tyrosine kinase.. Clin Cancer Res.

[146] Hamilton DH, Litzinger MT, Jales A, Huang B, Fernando RI (2013). Immunological targeting of tumor cells undergoing an epithelial-mesenchymal transition via a recombinant brachyury-yeast vaccine.. Oncotarget.

[147] Heery CR, Singh BH, Rauckhorst M, Marté JL, Donahue RN (2015). Phase I trial of a yeast-based therapeutic cancer vaccine (GI-6301) targeting the transcription factor brachyury.. Cancer Immunol Res.

[148] Heery CR, Palena C, McMahon S, Donahue RN, Lepone LM (2017). Phase I study of a poxviral TRICOM-based vaccine directed against the transcription factor brachyury.. Clin Cancer Res.

[149] Gatti-Mays ME, Redman JM, Donahue RN, Palena C, Madan RA (2019). A phase I trial using a multitargeted recombinant adenovirus 5 (CEA/MUC1/Brachyury)-based immunotherapy vaccine regimen in patients with advanced cancer.. Oncologist.

[150] van Kooten C, Banchereau J (2000). CD40-CD40 ligand.. J Leukoc Biol.

[151] Byrne KT, Vonderheide RH (2016). CD40 Stimulation Obviates Innate Sensors and Drives T Cell Immunity in Cancer.. Cell Rep.

[152] Vonderheide RH, Flaherty KT, Khalil M, Stumacher MS, Bajor DL (2007). Clinical activity and immune modulation in cancer patients treated with CP-870,893, a novel CD40 agonist monoclonal antibody.. J Clin Oncol.

[153] Croft M (2010). Control of immunity by the TNFR-related molecule OX40 (CD134).. Annu Rev Immunol.

[154] Curti BD, Kovacsovics-Bankowski M, Morris N, Walker E, Chisholm L (2013). OX40 is a potent immune-stimulating target in late-stage cancer patients.. Cancer Res.

[155] Sugamura K, Ishii N, Weinberg AD (2004). Therapeutic targeting of the effector T-cell co-stimulatory molecule OX40.. Nat Rev Immunol.

[156] Redmond WL, Linch SN, Kasiewicz MJ (2014). Combined targeting of costimulatory (OX40) and coinhibitory (CTLA-4) pathways elicits potent effector T cells capable of driving robust antitumor immunity.. Cancer Immunol Res.

[157] Topalian SL, Drake CG, Pardoll DM (2015). Immune checkpoint blockade: a common denominator approach to cancer therapy.. Cancer Cell.

[158] Fourcade J, Sun Z, Benallaoua M, Guillaume P, Luescher IF (2010). Upregulation of Tim-3 and PD-1 expression is associated with tumor antigen-specific CD8+ T cell dysfunction in melanoma patients.. J Exp Med.

[159] Thommen DS, Schreiner J, Muller P, Herzig P, Roller A (2015). Progression of lung cancer is associated with increased dysfunction of T cells defined by coexpression of multiple inhibitory receptors.. Cancer Immunol Res.

[160] Granier C, Dariane C, Combe P, Verkarre V, Urien S (2017). Tim-3 expression on tumor-infiltrating PD-1(+)CD8(+) T cells correlates with poor clinical outcome in renal cell carcinoma.. Cancer Res.

[161] Johnston RJ, Comps-Agrar L, Hackney J, Yu X, Huseni M (2014). The immunoreceptor TIGIT regulates antitumor and antiviral CD8(+) T cell effector function.. Cancer Cell.

[162] Kurtulus S, Sakuishi K, Ngiow SF, Joller N, Tan DJ (2015). TIGIT predominantly regulates the immune response via regulatory T cells.. J Clin Invest.

[163] Solomon BL, Garrido-Laguna I (2018). TIGIT: a novel immunotherapy target moving from bench to bedside.. Cancer Immunol Immunother.

[164] Liu W, Tang L, Zhang G, Wei H, Cui Y (2004). Characterization of a novel C-type lectin-like gene, LSECtin: demonstration of carbohydrate binding and expression in sinusoidal endothelial cells of liver and lymph node.. J Biol Chem.

[165] Kouo T, Huang L, Pucsek AB, Cao M, Solt S (2015). Galectin-3 shapes antitumor immune responses by suppressing CD8+ T cells via LAG-3 and inhibiting expansion of plasmacytoid dendritic cells.. Cancer Immunol Res.

[166] Jha V, Workman CJ, McGaha TL, Li L, Vas J (2014). Lymphocyte activation gene-3 (LAG-3) negatively regulates environmentally-induced autoimmunity.. PLoS One.

[167] Ascierto PA, Bono P, Bhatia S, Melero I, Nyakas MS (2017). LBA18Efficacy of BMS-986016, a monoclonal antibody that targets lymphocyte activation gene-3 (LAG-3), in combination with nivolumab in pts with melanoma who progressed during prior anti-PD-1/PD-L1 therapy (mel prior IO) in all-comer and biomarker-enriched populations.. Ann Oncol.

[168] Chon SY, Hassanain HH, Gupta SL (1996). Cooperative role of interferon regulatory factor 1 and p91 (STAT1) response elements in interferon-gamma-inducible expression of human indoleamine 2,3-dioxygenase gene.. J Biol Chem.

[169] Yentz S, Smith D (2018). Indoleamine 2,3-Dioxygenase (IDO) Inhibition as a strategy to augment cancer immunotherapy.. BioDrugs.

[170] Smith C, Chang MY, Parker KH, Beury DW, DuHadaway JB (2012). IDO is a nodal pathogenic driver of lung cancer and metastasis development.. Cancer Discov.

[171] Beatty GL, O’Dwyer PJ, Clark J, Shi JG, Bowman KJ (2017). First-in-Human Phase I study of the oral inhibitor of indoleamine 2,3-Dioxygenase-1 epacadostat (INCB024360) in patients with advanced solid malignancies.. Clin Cancer Res.

[172] Mitchell TC, Hamid O, Smith DC, Bauer TM, Wasser JS (2018). Epacadostat plus pembrolizumab in patients with advanced solid tumors: phase i results from a multicenter, open-label phase I/II trial (ECHO-202/KEYNOTE-037).. J Clin Oncol.

[173] Long GV, Dummer R, Hamid O, Gajewski TF, Caglevic C (2019). Epacadostat plus pembrolizumab versus placebo plus pembrolizumab in patients with unresectable or metastatic melanoma (ECHO-301/KEYNOTE-252): a phase 3, randomised, double-blind study.. Lancet Oncol.

[174] Kerbel RS (2008). Tumor angiogenesis.. N Engl J Med.

[175] Motz GT, Santoro SP, Wang LP, Garrabrant T, Lastra RR (2014). Tumor endothelium FasL establishes a selective immune barrier promoting tolerance in tumors.. Nat Med.

[176] Chen PL, Roh W, Reuben A, Cooper ZA, Spencer CN (2016). Analysis of immune signatures in longitudinal tumor samples yields insight into biomarkers of response and mechanisms of resistance to immune checkpoint blockade.. Cancer Discov.

[177] Voron T, Colussi O, Marcheteau E, Pernot S, Nizard M (2015). VEGF-A modulates expression of inhibitory checkpoints on CD8+ T cells in tumors.. J Exp Med.

[178] Ohm JE, Gabrilovich DI, Sempowski GD, Kisseleva E, Parman KS (2003). VEGF inhibits T-cell development and may contribute to tumor-induced immune suppression.. Blood.

[179] Terme M, Pernot S, Marcheteau E, Sandoval F, Benhamouda N (2013). VEGFA-VEGFR pathway blockade inhibits tumor-induced regulatory T-cell proliferation in colorectal cancer.. Cancer Res.

[180] Yasuda S, Sho M, Yamato I, Yoshiji H, Wakatsuki K (2013). Simultaneous blockade of programmed death 1 and vascular endothelial growth factor receptor 2 (VEGFR2) induces synergistic anti-tumour effect in vivo.. Clin Exp Immunol.

[181] Wallin JJ, Bendell JC, Funke R, Sznol M, Korski K (2016). Atezolizumab in combination with bevacizumab enhances antigen-specific T-cell migration in metastatic renal cell carcinoma.. Nat Commun.

[182] Bergerot P, Lamb P, Wang E, Pal SK (2019). Cabozantinib in combination with immunotherapy for advanced renal cell carcinoma and urothelial carcinoma: rationale and clinical evidence.. Mol Cancer Ther.

[183] Rini BI, Plimack ER, Stus V, Gafanov R, Hawkins R (2019). Pembrolizumab plus axitinib versus sunitinib for advanced renal-cell carcinoma.. N Engl J Med.

[184] Motzer RJ, Penkov K, Haanen J, Rini B, Albiges L (2019). Avelumab plus axitinib versus sunitinib for advanced renal-cell carcinoma.. N Engl J Med.

[185] Hsu C-H, Lee MS, Lee KH, Numata K, Stein S (2019). LBA7Randomised efficacy and safety results for atezolizumab (Atezo) + bevacizumab (Bev) in patients (pts) with previously untreated, unresectable hepatocellular carcinoma (HCC).. Ann Oncol.

[186] Lane ER, Zisman TL, Suskind DL (2017). The microbiota in inflammatory bowel disease: current and therapeutic insights.. J Inflamm Res.

[187] Zitvogel L, Daillere R, Roberti MP, Routy B, Kroemer G (2017). Anticancer effects of the microbiome and its products.. Nat Rev Microbiol.

[188] Zitvogel L, Ayyoub M, Routy B, Kroemer G (2016). Microbiome and anticancer immunosurveillance.. Cell.

[189] Fessler J, Matson V, Gajewski TF (2019). Exploring the emerging role of the microbiome in cancer immunotherapy.. J Immunother Cancer.

[190] Gopalakrishnan V, Spencer CN, Nezi L, Reuben A, Andrews MC (2018). Gut microbiome modulates response to anti-PD-1 immunotherapy in melanoma patients.. Science.

[191] Matson V, Fessler J, Bao R, Chongsuwat T, Zha Y (2018). The commensal microbiome is associated with anti-PD-1 efficacy in metastatic melanoma patients.. Science.

[192] Routy B, Le Chatelier E, Derosa L, Duong CPM, Alou MT (2018). Gut microbiome influences efficacy of PD-1-based immunotherapy against epithelial tumors.. Science.

[193] Vetizou M, Pitt JM, Daillere R, Lepage P, Waldschmitt N (2015). Anticancer immunotherapy by CTLA-4 blockade relies on the gut microbiota.. Science.

[194] Derosa L, Hellmann MD, Spaziano M, Halpenny D, Fidelle M (2018). Negative association of antibiotics on clinical activity of immune checkpoint inhibitors in patients with advanced renal cell and non-small-cell lung cancer.. Ann Oncol.

[195] Pinato DJ, Howlett S, Ottaviani D, Urus H, Patel A (2019). Association of prior antibiotic treatment with survival and response to immune checkpoint inhibitor therapy in patients with cancer.. JAMA Oncol.

[196] Sivan A, Corrales L, Hubert N, Williams JB, Aquino-Michaels K (2015). Commensal bifidobacterium promotes antitumor immunity and facilitates anti-PD-L1 efficacy.. Science.

[197] Baruch EN, Youngster I, Ortenberg R, Ben-Betzalel G, Katz LH (2019). Abstract CT042: fecal microbiota transplantation (FMT) and re-induction of anti-PD-1 therapy in refractory metastatic melanoma patients - preliminary results from a phase I clinical trial (NCT03353402).. Cancer Res.

[198] Butterfield LH, Disis ML, Fox BA, Kaufman DR, Khleif SN (2018). SITC 2018 workshop report: immuno-oncology biomarkers: state of the art.. J Immunother Cancer.

[199] Balli D, Rech AJ, Stanger BZ, Vonderheide RH (2017). Immune cytolytic activity stratifies molecular subsets of human pancreatic cancer.. Clin Cancer Res.

[200] Blando J, Sharma A, Higa MG, Zhao H, Vence L (2019). Comparison of immune infiltrates in melanoma and pancreatic cancer highlights VISTA as a potential target in pancreatic cancer.. Proc Natl Acad Sci U S A.

[201] Lee JJ, Powderly JD, Patel MR, Brody J, Hamilton EP (2017). Phase 1 trial of CA-170, a novel oral small molecule dual inhibitor of immune checkpoints PD-1 and VISTA, in patients (pts) with advanced solid tumor or lymphomas.. J Clin Oncol.

[202] Tolcher AW, Sznol M, Hu-Lieskovan S, Papadopoulos KP, Patnaik A (2017). Phase Ib study of utomilumab (PF-05082566), a 4-1BB/CD137 agonist, in combination with pembrolizumab (MK-3475) in patients with advanced solid tumors.. Clin Cancer Res.

[203] Soldevilla MM, Villanueva H, Meraviglia-Crivelli D, Menon AP, Ruiz M (2019). ICOS costimulation at the tumor site in combination with CTLA-4 blockade therapy elicits strong tumor immunity.. Mol Ther.

[204] Zappasodi R, Sirard C, Li Y, Budhu S, Abu-Akeel M (2019). Rational design of anti-GITR-based combination immunotherapy.. Nat Med.

[205] Suzuki E, Kapoor V, Jassar AS, Kaiser LR, Albelda SM (2005). Gemcitabine selectively eliminates splenic Gr-1+/CD11b+ myeloid suppressor cells in tumor-bearing animals and enhances antitumor immune activity.. Clin Cancer Res.

[206] Germano G, Lamba S, Rospo G, Barault L, Magrì A (2017). Inactivation of DNA repair triggers neoantigen generation and impairs tumour growth.. Nature.

[207] Wakita D, Iwai T, Harada S, Suzuki M, Yamamoto K (2019). Cisplatin augments antitumor T-cell responses leading to a potent therapeutic effect in combination with PD-L1 blockade.. Anticancer Res.

[208] Zhu L, Chen L (2019). Progress in research on paclitaxel and tumor immunotherapy.. Cell Mol Biol Lett.

[209] Ganesh S, Shui X, Craig KP, Park J, Wang W (2018). RNAi-mediated β-catenin inhibition promotes T cell infiltration and antitumor activity in combination with immune checkpoint blockade.. Mol Ther.

[210] Kawazoe A, Kuboki Y, Komatsu Y, Nishina T, Shinozaki E (2018). Multicenter phase I/II trial of BBI608 and pembrolizumab combination in patients with metastatic colorectal cancer (SCOOP Study): EPOC1503.. J Clin Oncol.

[211] Luo N, Formisano L, Gonzalez-Ericsson PI, Sanchez V, Dean PT (2018). Melanoma response to anti-PD-L1 immunotherapy requires JAK1 signaling, but not JAK2.. Oncoimmunology.

[212] Schaer DA, Beckmann RP, Dempsey JA, Huber L, Forest A (2018). The CDK4/6 inhibitor abemaciclib induces a T cell inflamed tumor microenvironment and enhances the efficacy of PD-L1 checkpoint blockade.. Cell Rep.

[213] Jiang P, Gu S, Pan D, Fu J, Sahu A (2018). Signatures of T cell dysfunction and exclusion predict cancer immunotherapy response.. Nat Med.

[214] Beavis PA, Milenkovski N, Henderson MA, John LB, Allard B (2015). Adenosine receptor 2A blockade increases the efficacy of anti-PD-1 through enhanced antitumor T-cell responses.. Cancer Immunol Res.

[215] Chen L, Diao L, Yang Y, Yi X, Rodriguez BL (2018). CD38-mediated immunosuppression as a mechanism of tumor cell escape from PD-1/PD-L1 blockade.. Cancer Discov.

[216] Maj T, Wang W, Crespo J, Zhang H, Wang W (2017). Oxidative stress controls regulatory T cell apoptosis and suppressor activity and PD-L1-blockade resistance in tumor.. Nat Immunol.

[217] Perrot I, Michaud HA, Giraudon-Paoli M, Augier S, Docquier A (2019). Blocking antibodies targeting the CD39/CD73 immunosuppressive pathway unleash immune responses in combination cancer therapies.. Cell Rep.

[218] Iannone R, Miele L, Maiolino P, Pinto A, Morello S (2014). Adenosine limits the therapeutic effectiveness of anti-CTLA4 mAb in a mouse melanoma model.. Am J Cancer Res.

[219] Leone RD, Sun IM, Oh MH, Sun IH, Wen J (2018). Inhibition of the adenosine A2a receptor modulates expression of T cell coinhibitory receptors and improves effector function for enhanced checkpoint blockade and ACT in murine cancer models.. Cancer Immunol Immunother.

[220] Doi T, Muro K, Ishii H, Kato T, Tsushima T (2019). A phase I study of the anti-CC chemokine receptor 4 antibody, mogamulizumab, in combination with nivolumab in patients with advanced or metastatic solid tumors.. Clin Cancer Res.

[221] Buchbinder EI, Dutcher JP, Daniels GA, Curti BD, Patel SP (2019). Therapy with high-dose Interleukin-2 (HD IL-2) in metastatic melanoma and renal cell carcinoma following PD1 or PDL1 inhibition.. J Immunother Cancer.

[222] Puca E, Probst P, Stringhini M, Murer P, Pellegrini G (2020). The antibody-based delivery of interleukin-12 to solid tumors boosts NK and CD8(+) T cell activity and synergizes with immune checkpoint inhibitors.. Int J Cancer.

[223] Berraondo P, Etxeberria I, Ponz-Sarvise M, Melero I (2018). Revisiting interleukin-12 as a cancer immunotherapy agent.. Clin Cancer Res.

[224] Zhao M, Luo M, Xie Y, Jiang H, Cagliero C (2019). Development of a recombinant human IL-15·sIL-15Rα/Fc superagonist with improved half-life and its antitumor activity alone or in combination with PD-1 blockade in mouse model.. Biomed Pharmacother.

[225] Jung H, Bischof A, Ebsworth K, Ertl L, Schall T (2015). Combination therapy of chemokine receptor inhibition plus PDL-1 blockade potentiates anti-tumor effects in a murine model of breast cancer.. J Immunother Cancer.

[226] Flores-Toro JA, Luo D, Gopinath A, Sarkisian MR, Campbell JJ (2020). CCR2 inhibition reduces tumor myeloid cells and unmasks a checkpoint inhibitor effect to slow progression of resistant murine gliomas.. Proc Natl Acad Sci U S A.

[227] Jung H, Ertl L, Janson C, Schall T, Charo I (2016). Abstract A107: Inhibition of CCR2 potentiates the checkpoint inhibitor immunotherapy in pancreatic cancer.. Cancer Immunol Res.

[228] Dominguez C, McCampbell KK, David JM, Palena C (2017). Neutralization of IL-8 decreases tumor PMN-MDSCs and reduces mesenchymalization of claudin-low triple-negative breast cancer.. JCI insight.

[229] Greene S, Robbins Y, Mydlarz WK, Huynh AP, Schmitt NC (2020). Inhibition of MDSC trafficking with SX-682, a CXCR1/2 inhibitor, enhances NK-Cell immunotherapy in head and neck cancer models.. Clin Cancer Res.

[230] Steele CW, Karim SA, Leach JDG, Bailey P, Upstill-Goddard R (2016). CXCR2 inhibition profoundly suppresses metastases and augments immunotherapy in pancreatic ductal adenocarcinoma.. Cancer Cell.

[231] Mullinax JE, Hall M, Prabhakaran S, Weber J, Khushalani N (2018). Combination of ipilimumab and adoptive cell therapy with tumor-infiltrating lymphocytes for patients with metastatic melanoma.. Front Oncol.

[232] Zhang R, Deng Q, Jiang YY, Zhu HB, Wang J (2019). Effect and changes in PD-1 expression of CD19 CAR-T cells from T cells highly expressing PD-1 combined with reduced-dose PD-1 inhibitor.. Oncol Rep.

[233] Peng M, Mo Y, Wang Y, Wu P, Zhang Y (2019). Neoantigen vaccine: an emerging tumor immunotherapy.. Molecular Cancer.

[234] Wang T, Zheng N, Luo Q, Jiang L, He B (2019). Probiotics lactobacillus reuteri abrogates immune checkpoint blockade-associated colitis by inhibiting group 3 innate lymphoid cells.. Front Immunol.

[235] Deng L, Liang H, Burnette B, Beckett M, Darga T (2014). Irradiation and anti-PD-L1 treatment synergistically promote antitumor immunity in mice.. J Clin Invest.

[236] Twyman-Saint Victor C, Rech AJ, Maity A, Rengan R, Pauken KE (2015). Radiation and dual checkpoint blockade activate non-redundant immune mechanisms in cancer.. Nature.

[237] Demaria S, Golden EB, Formenti SC (2015). Role of local radiation therapy in cancer immunotherapy.. JAMA Oncol.

[238] Lemdani K, Mignet N, Boudy V, Seguin J, Oujagir E (2019). Local immunomodulation combined to radiofrequency ablation results in a complete cure of local and distant colorectal carcinoma.. Oncoimmunology.

[239] Aarts BM, Klompenhouwer EG, Rice SL, Imani F, Baetens T (2019). Cryoablation and immunotherapy: an overview of evidence on its synergy.. Insights into Imaging.

[240] Ray A, Williams MA, Meek SM, Bowen RC, Grossmann KF (2016). A phase I study of intratumoral ipilimumab and interleukin-2 in patients with advanced melanoma.. Oncotarget.

[241] Weide B, Derhovanessian E, Pflugfelder A, Eigentler TK, Radny P (2010). High response rate after intratumoral treatment with interleukin-2: results from a phase 2 study in 51 patients with metastasized melanoma.. Cancer.

[242] Langan EA, Kümpers C, Graetz V, Perner S, Zillikens D (2019). Intralesional interleukin-2: a novel option to maximize response to systemic immune checkpoint therapy in loco-regional metastatic melanoma.. Dermatol Ther.

[243] Rafei-Shamsabadi D, Lehr S, von Bubnoff D, Meiss F (2019). Successful combination therapy of systemic checkpoint inhibitors and intralesional interleukin-2 in patients with metastatic melanoma with primary therapeutic resistance to checkpoint inhibitors alone.. Cancer Immunol Immunother.

[244] Chowdhury S, Castro S, Coker C, Hinchliffe TE, Arpaia N (2019). Programmable bacteria induce durable tumor regression and systemic antitumor immunity.. Nat Med.

[245] Marabelle A, Tselikas L, De Baere T, Houot R (2017). Intratumoral immunotherapy: using the tumor as the remedy.. Ann Oncol.

[246] Wang Y, Liu J, Yang X, Liu Y, Liu Y (2018). Bacillus Calmette-Guérin and anti-PD-L1 combination therapy boosts immune response against bladder cancer.. Onco Targets Ther.

